# Lute-Gen^®^ Alleviates Dry Eye Disease and Modulates Nrf2/HO-1, TLR4/NF-κB/MAPK Signaling, and Aquaporin-Mediated Tear Homeostasis

**DOI:** 10.3390/antiox15070872

**Published:** 2026-07-13

**Authors:** Rachit Sood, Sanjay, Hae-Jeung Lee

**Affiliations:** 1 Department of Health Sciences and Technology, Gachon Advanced Institute for Health Science and Technology (GAIHST), Gachon University, Incheon 21999, Republic of Korea; soodrachit@gachon.ac.kr; 2 Institute for Aging and Clinical Nutrition Research, Gachon University, Seongnam 13120, Republic of Korea; 3 Department of Food and Nutrition, College of BioNano Technology, Gachon University, Seongnam 13120, Republic of Korea; 4 Gachon Biomedical Convergence Institute, Gachon University Gil Medical Center, Incheon 21565, Republic of Korea

**Keywords:** dry eye disease, lutein and zeaxanthin, oxidative stress, inflammation, ocular surface homeostasis, scopolamine-induced model

## Abstract

Dry eye disease (DED) is a multifactorial ocular surface disorder characterized by inflammation, oxidative stress, tear film instability, and secretory dysfunction. This study investigated the protective effects of Lute-gen^®^, a lutein and zeaxanthin-based formulation, in both in vitro and in vivo DED models. Human corneal epithelial (HCE-T) cells were stimulated with TNF-α, while dry eye was induced in female Sprague–Dawley rats using subcutaneous scopolamine (SCP) administration. In HCE-T cells, Lute-gen^®^ showed no cytotoxicity, restored cell viability, reduced intracellular ROS, and was associated with increased expression of antioxidant-related markers (Nrf2, HO-1, SOD, CAT, and GPx), reduced expression of inflammatory mediators (TLR4/MyD88/NF-κB/NLRP3), and increased expression of AQP3 and AQP5. In SCP-induced rats, Lute-gen^®^ significantly improved tear secretion and reduced corneal fluorescein staining. Histopathological analyses revealed restoration of conjunctival goblet cells, mucin staining, corneal epithelial integrity, acinar area and cell density, and lacrimal gland architecture, with reduced inflammatory infiltration. Immunofluorescence further demonstrated reduced TLR4 and MMP9 immunoreactivity and decreased CD68^+^ inflammatory cell infiltration. Molecular analyses showed reduced expression of inflammatory cytokines and NF-κB/MAPK/MMP signaling-related inflammatory mediators, together with restoration of AQP1, AQP3, and AQP5 expression in corneal tissues. Collectively, these findings suggest that Lute-gen^®^ treatment was associated with improvements in dry eye-related pathological changes, including restoration of antioxidant-related markers, attenuation of inflammatory responses, restoration of aquaporin expression, and preservation of ocular surface and lacrimal gland integrity. These preclinical findings support further mechanistic investigations and the future clinical evaluation of Lute-gen^®^ as a potential nutritional intervention for dry eye disease.

## 1. Introduction

Dry eye disease (DED) is a common, multifactorial disorder of the ocular surface in which loss of tear-film homeostasis drives a self-perpetuating cycle of tear instability, hyperosmolar stress, inflammation, tissue injury, and neurosensory disturbance [1]. The condition is often accompanied by symptoms such as dryness, burning, foreign body sensation, and visual disturbance, which significantly impair quality of life [2]. Epidemiologic analyses from the TFOS DEWS II reports estimate that DED affects approximately 5–50% of the global population, and its prevalence continues to increase due to aging, environmental stress, and increased digital screen use [2,3].

Although DED has diverse etiologies, disruption of ocular surface homeostasis is a central mechanism driving disease progression. Tear film instability and hyperosmolar stress initiate inflammatory cascades that result in epithelial damage and further destabilization of the tear film [4]. Pro-inflammatory cytokines, particularly tumor necrosis factor-α (TNF-α), play a key role in disrupting epithelial barrier integrity and promoting downstream inflammatory signaling in corneal epithelial cells [5]. In addition, increased expression of matrix metalloproteinases (MMP2 and MMP9) contributes to extracellular matrix degradation and epithelial injury, thereby further exacerbating disease severity [6,7]. Moreover, the infiltration of inflammatory immune cells, particularly macrophages, has been reported in the ocular surface of DED, where these cells contribute to cytokine production and tissue damage. Accordingly, the cluster of differentiation 68 (CD68) is widely used as a marker of macrophage-associated inflammation in experimental models [8,9].

Oxidative stress (OS) is another critical contributor to DED pathogenesis. The accumulation of reactive oxygen species (ROS) has been reported in both clinical and experimental dry eye conditions, leading to cellular damage and apoptosis in ocular surface tissues [10,11]. The nuclear factor erythroid 2-related factor 2 (Nrf2) pathway serves as a major cellular defense mechanism against OS by regulating antioxidant gene expression [12]. Activation of Nrf2 has been shown to protect corneal epithelial cells and ameliorate dry eye symptoms in both in vitro and in vivo models [13,14]. Furthermore, inflammatory signaling pathways, including nuclear factor kappa B (NF-κB) and the mitogen-activated protein kinase (MAPK) pathways, particularly extracellular signal-regulated kinase (ERK) and p38, are closely linked to OS and regulate cytokine production and apoptotic responses in dry eye conditions [15,16]. In addition, the activation of these pathways promotes immune cell recruitment and activation, further amplifying ocular surface inflammation [15].

Beyond the cornea, dysfunction of tear-secreting tissues also plays a critical role in DED. Loss of conjunctival goblet cells leads to reduced mucin production, impairing tear film stability and ocular surface lubrication [17,18]. Similarly, structural and functional alterations in the lacrimal gland result in decreased aqueous tear secretion and increased inflammatory infiltration [19,20,21]. Aquaporins, which regulate transmembrane water transport across epithelial tissues, have also been implicated in maintaining tear secretion and ocular surface hydration, and their dysregulation has been implicated in dry eye pathology [22].

Current treatments for DED, including artificial tears and anti-inflammatory drugs, primarily provide symptomatic relief and are often associated with limited efficacy or adverse effects during long-term use [3]. Therefore, there is a growing interest in developing safe and effective intervention strategies targeting multiple pathological processes of DED, including OS, inflammation, and tear film dysfunction. Lutein and zeaxanthin are dietary xanthophyll carotenoids with well-established antioxidant and anti-inflammatory properties, and have been extensively investigated for maintaining ocular health, particularly in retinal disorders [23,24]. Increasing evidence suggests that these carotenoids may also modulate redox-sensitive and inflammatory signaling pathways that are central to cellular stress responses, including those relevant to DED [23]. In corneal epithelial cells, lutein has been shown to attenuate hyperosmolarity-induced inflammatory responses, including suppression of interleukin (IL)-6 expression through inhibition of p38, c-Jun N-terminal kinase (JNK), and NF-κB signaling pathways [25]. Beyond ocular-specific models, lutein has also been reported to enhance cellular antioxidant defense mechanisms, primarily through activation of the Nrf2/heme oxygenase (HO)-1 signaling pathway in an osteoporosis rat model, thereby contributing to protection against ROS-mediated cellular damage [26]. Similarly, zeaxanthin has demonstrated protective effects against oxidative and UV-induced cellular damage in human ocular surface epithelial cells and has been shown to enhance antioxidant capacity through the activation of Nrf2-mediated antioxidant responses in HepG2 human hepatocellular carcinoma cells, supporting its broader role in protection against OS [27,28]. Clinical studies have also reported improvements in selected dry eye-related parameters following supplementation with formulations containing lutein and zeaxanthin; however, these studies have primarily evaluated multi-component dietary supplements, making it difficult to determine the specific contribution of lutein and zeaxanthin to the observed effects [29].

Despite these findings, important knowledge gaps remain regarding the therapeutic potential of standardized lutein–zeaxanthin formulations in DED. Most previous experimental studies have evaluated lutein or zeaxanthin individually, whereas the limited available clinical studies have primarily investigated multi-component dietary formulations, making it difficult to define the specific contributions of lutein and zeaxanthin to DED pathology [25,27,29]. In addition, mechanistic investigations examining whether a standardized lutein–zeaxanthin formulation with a defined composition can improve ocular surface integrity, tear film function, and regulate OS- and inflammation-related signaling pathways in experimental DED models have not been comprehensively investigated. Therefore, the present study aimed to investigate the protective efficacy of Lute-gen^®^ using complementary in vitro and in vivo DED models by comprehensively assessing tear secretion, corneal epithelial integrity, OS, inflammatory signaling, and histopathological alterations. By integrating functional, molecular, and histopathological analyses, this study provides a more comprehensive evaluation of the therapeutic potential and underlying mechanisms of a standardized lutein–zeaxanthin formulation in DED.

Specifically, an in vitro model using TNF-α-stimulated human corneal epithelial (HCE-T) cells was employed to evaluate inflammatory and oxidative responses, whereas an in vivo scopolamine (SCP)-induced rat model was used to mimic aqueous-deficient dry eye. The effects of Lute-gen^®^ treatment on key functional, molecular, and histopathological changes associated with DED, including tear secretion, inflammatory signaling-, antioxidant defense-, and matrix degradation-related markers, together with aquaporin expression, and histopathological alterations in the conjunctiva, cornea, and lacrimal gland were comprehensively evaluated.

## 2. Materials and Methods

### 2.1. Materials

The human corneal epithelial HCE-T cells (305255) and Dulbecco’s modified Eagle’s medium/Ham’s F-12 (DMEM/F-12, 1:1) used in this study were obtained from Cytion (Heidelberg, Germany). The primary monoclonal antibodies for Nrf2 (sc-365949), HO-1 (sc-136960), superoxide dismutase 1 (SOD1, sc-101523), glutathione peroxidase 1/2 (GPx1/2, sc-133160), toll-like receptor-4 (TLR4, sc-293072), myeloid differentiation primary response 88 (MyD88, sc-74532), aquaporin-1 (AQP-1, sc-25287), AQP3 (sc-518001), AQP5 (sc-514022), MMP2 (sc-13595), MMP9 (sc-393859), CD68 (sc-20060), and β-actin (sc-47778, 1:5000) were obtained from Santa Cruz Biotechnology (Dallas, TX, USA). OriGene (Rockville, MD, USA) supplied the monoclonal antibody for catalase (CAT, CF502564). The primary antibodies for phosphorylated-NF-κB (pNF-κB, S536), NF-κB (D14E12), nod-like receptor family, pyrin domain-containing protein 3 (NLRP3, D4D8T), p-p38 MAPK (Thr180/Tyr182, 4511S), p38 MAPK (8690S), p-ERK1/2 (p-p44/42 MAPK, Thr202/Tyr204, 4370S), and ERK1/2 (p44/42 MAPK, 4695S) were procured from Cell Signaling Technology (Danvers, MA, USA). The recombinant human epidermal growth factor protein (EGF, PHG0311), insulin–transferrin–selenium–ethanolamine (ITS-X, 51500-056), fetal bovine serum (FBS, 16000-044), and anti-anti solution (15240-062) were purchased from ThermoFisher Scientific (Waltham, MA, USA). Recombinant human TNF-α (210-TA) was obtained from R&D Systems (Minneapolis, MN, USA). Scopolamine hydrobromide (SCP, S0021) was procured from Tokyo Chemical Industry (Tokyo, Japan). The standard rodent chow was sourced from LabAnimal, Korea Science (Seoul, Republic of Korea).

Lute-gen^®^, a marigold (*Tagetes erecta*) flower extract, was supplied by Bio-gen Extracts Pvt. Ltd. (Bangalore, India) through FINE BS Co., Ltd. (Seoul, Republic of Korea) and used as the test substance in the present study. The same Lute-gen^®^ ingredient was previously evaluated in a human clinical trial [30].

### 2.2. Lute-Gen^®^ Characterization

Lute-gen^®^ is an oil-based formulation manufactured by Bio-gen Extracts Pvt. Ltd., India, and standardized to contain 20–24% lutein and ≥4% zeaxanthin. The formulation contains a sunflower oil matrix with D-α-tocopherol as an excipient. Batch-specific third-party HPLC analysis verified lutein and zeaxanthin contents of 203.36 mg/g and 48.26 mg/g, respectively. The product is manufactured under ISO 9001 [31], ISO 22000 [32], and Good Manufacturing Practice (GMP) certified quality management systems, and the production batch met the manufacturer’s quality specifications, including testing for heavy metals, mycotoxins, pesticide residues, microbial contamination, and residual solvents.

### 2.3. Cell Culture

The human corneal epithelial HCE-T cells were maintained in DMEM/F-12, 1:1 supplemented with 5% FBS, 1% ITS-X (0.625 mg/mL human insulin, 0.625 mg/mL human transferrin, 0.625 microgram/mL sodium selenite, 0.535 mg/mL linoleic acid, 125 mg/mL BSA), 10 ng/mL human EGF and 1X anti-anti solution in a humidified incubator with 5% CO_2_ at 37 °C.

### 2.4. Cell Viability Assay

HCE-T cells (1 × 10^4^ cells/well) were seeded and allowed to adhere for 24 h in 96-well plates. The cells were exposed to various concentrations of Lute-gen^®^ (1.25, 2.5, 5, 7.5, 10, 15, 20, 40, 60, 80, and 100 μg/mL) or TNF-α (0.5, 1, 2.5, 5, 7.5, 10, 12.5, 15, 17.5, and 20 ng/mL) for 24 h or co-treated with TNF-α (10 ng/mL) and Lute-gen^®^ (2.5, 5, 7.5, 10, 15, 20, 40, 60, 80, and 100 μg/mL) for 24 h at 37 °C under 5% CO_2_ atmosphere. Cell viability was determined using the Cell Counting Kit-8 assay (Dojindo Molecular Technologies, Rockville, MD, USA) according to the manufacturer’s instructions. The absorbance was measured at 450 nm using a microplate reader (BioTek, Winooski, VT, USA).

### 2.5. Experimental Animals

Sixty female Sprague–Dawley (SD) rats were procured from Daehan Biolink Co., Eumseong, Republic of Korea. The animals were housed under controlled environmental conditions with a temperature of 20–25 °C, relative humidity of 50–60%, and a 12 h light/dark cycle. All rats were allowed to acclimatize for 7 days prior to the initiation of the experiment. Standard laboratory chow and water were provided ad libitum throughout the study period. All experimental procedures were approved by the Institutional Animal Care and Use Committee (IACUC) of Gachon University (GU1-2025-IA0013) and conducted in accordance with the Guide for the Care and Use of Laboratory Animals.

### 2.6. Induction of Dry Eye Animal Model

**Figure 1:**
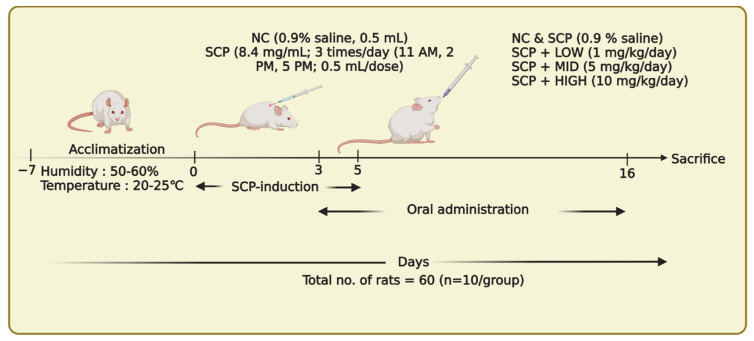
Experimental Setup. Dry eye disease was induced in SD rats by subcutaneous injection of SCP (8.4 mg/mL, 0.5 mL per injection) three times daily (11:00, 14:00, and 17:00) for 5 consecutive days. From day 3 of SCP administration, rats received oral treatments once daily until day 16. Treatment groups included LOW, MID, and HIGH concentrations of Lute-gen^®^ at 1, 5, and 10 mg/kg/day, respectively. The NC group received saline only. Animals were sacrificed on day 16 following the treatment period. NC, normal control; SCP, scopolamine-treated dry eye model; LOW, Lute-gen^®^ (1 mg/kg); MID, Lute-gen^®^ (5 mg/kg); and HIGH, Lute-gen^®^ (10 mg/kg). Created in BioRender. Lee, H. (2026) https://BioRender.com/46e7zgi (accessed on 2 July 2026).

After a one-week acclimatization period, the rats were weighed and randomly assigned to five experimental groups such that the mean body weights were comparable (~137.4 ± 6.8 g) across groups (*n* = 10 per group): NC (normal control), SCP (DED control), LOW (SCP + low-dose Lute-gen^®^), MID (SCP + mid-dose Lute-gen^®^), and HIGH (SCP + high-dose Lute-gen^®^). DED was induced by subcutaneous injection of SCP (8.4 mg/mL dissolved in 0.9% saline) for 5 consecutive days. SCP was administered three times daily at 11:00 a.m., 2:00 p.m., and 5:00 p.m., with 0.5 mL per injection, from day 0 to day 5, to mimic a short-term DED model. The NC group received an equivalent volume of 0.9% saline following the same schedule. Oral administration of the respective treatments began on day 3 of SCP injection and continued for 14 consecutive days (day 3 to day 16), resulting in a total treatment duration of 14 days. The LOW, MID, and HIGH treatment groups received Lute-gen^®^ at doses of 1, 5, and 10 mg/kg/day, respectively. The selected dose range was based on the clinically used human intake of Lute-gen^®^ reported in a previous randomized clinical trial and converted to the rat-equivalent dose using the standard body surface area-based interspecies dose conversion method described in the FDA guidance [30,33]. Based on this calculation, 5 mg/kg/day corresponds to the clinically relevant rat-equivalent dose, whereas 1 mg/kg/day and 10 mg/kg/day were selected to evaluate the effects across a lower and higher exposure range, respectively. All oral administrations were performed once daily using oral gavage. The NC and SCP groups received an equivalent volume of vehicle solutions (0.9% saline). Throughout the experimental period, body weight, tear secretion, and general clinical signs were monitored. At the end of the treatment period, animals were sacrificed under appropriate anesthesia, and ocular tissues were collected for subsequent biochemical, histological, and molecular analyses. Corneal tissues from three randomly selected animals per group were used for RT-PCR analysis, whereas corneal tissues from six randomly selected animals per group were used for Western blot analysis. Ocular tissues from three randomly selected animals per group were processed for corneal fluorescein staining, histological evaluation, and immunofluorescence analyses. Different subsets of randomly selected animals were allocated to each experimental endpoint according to tissue requirements and the predefined experimental design. No predefined exclusion criteria were established before the study, and no animals or samples were excluded from the analyses. The sample sizes used for each experimental endpoint were selected based on previous studies using the SCP-induced dry eye model and tissue requirements for the respective analyses. The experimental design is shown in Figure 1.

### 2.7. Quantitative Real-Time PCR (RT-PCR) Analysis

For in vitro experiments, HCE-T cells were seeded in 6-well plates at a density of 1 × 10^6^ cells per well and allowed to attach for 24 h. Cells were then treated with 10 ng/mL of TNF-α in the presence or absence of Lute-gen^®^ (2.5, 5, and 7.5 μg/mL) for 24 h. After treatment, total RNA was extracted using a commercial RNA isolation kit (iNTRON Biotechnology, Seongnam, Republic of Korea) according to the manufacturer’s instructions. The extracted RNA was reverse-transcribed into complementary DNA (cDNA) using a PCR system (TaKaRa Bio, Shiga, Japan), and quantitative real-time PCR was performed using a SYBR Green-based master mix (TaKaRa Bio, Shiga, Japan) on an ABI Quant Studio 3 PCR system (Applied Biosystems, Foster City, CA, USA). For in vivo analysis, randomly selected animals from each group were allocated for RT-PCR analysis, and corneal tissues (*n* = 3 rats per group) were carefully harvested for total RNA extraction using the same protocol. The relative gene expression levels were standardized using the β-actin gene as an internal control, which exhibited stable Ct values across all experimental groups in this study. Specific primer sets for the target genes are listed in Table 1.

### 2.8. 2′,7′-Dichlorodihydrofluorescein Diacetate (DCFH-DA/H2DCFDA) Assay

Intracellular ROS levels were measured using the DCFH-DA assay. HCE-T cells were seeded at a density of 3 × 10^5^ cells per well in 6-well plates and incubated for 24 h at 37 °C in a humidified atmosphere containing 5% CO_2_. Following incubation, cells were treated with TNF-α (10 ng/mL) in the presence or absence of Lute-gen^®^ (2.5, 5, and 7.5 μg/mL) for 24 h. After treatment, cells were washed with phosphate-buffered saline (PBS) and incubated with 10 μM DCFH-DA for 30 min at 37 °C in the dark. Excess probe was removed by washing with PBS. Fluorescence images were captured using a fluorescence microscope (Olympus, Tokyo, Japan). The fluorescence intensity of DCF was quantified using ImageJ software version 1.54s (NIH, USA), and the results were expressed as relative fluorescence intensity normalized to the control group.

### 2.9. Western Blot Analysis

For in vitro experiments, HCE-T cells were seeded in 6-well plates and allowed to adhere for 24 h. Cells were then treated with 10 ng/mL of TNF-α in the presence or absence of Lute-gen^®^ (2.5, 5, and 7.5 μg/mL) for 24 h. After treatment, total cellular proteins were extracted using lysis buffer (iNtRON Biotechnology, Gyeonggi-do, Republic of Korea) supplemented with protease and phosphatase inhibitors. For in vivo analysis, corneal tissues were collected from experimental rats (*n* = 6 per group), homogenized, and lysed using the same lysis buffer containing protease and phosphatase inhibitors. The protein concentration was determined using the Bradford assay. Equal amounts of protein (40 μg) were separated by SDS-PAGE and transferred onto PVDF membranes (Bio-Rad Laboratories, Hercules, CA, USA). The membranes were blocked with 5% skim milk for 1 h at room temperature and incubated overnight at 4 °C with primary antibodies against antioxidant-related proteins (Nrf2- 1:500, HO-1- 1:500, SOD- 1:500, CAT- 1:500, and GPx- 1:500), inflammatory signaling molecules (TLR4- 1:500, MyD88- 1:500, NF-κB- 1:1000, phospho-NF-κB- 1:1000, NLRP3- 1:1000, MMP2- 1:500, MMP9- 1:500, ERK- 1:1000, phospho-ERK- 1:1000, p38- 1:1000, and phospho-p38- 1:1000), and aquaporins (AQP1- 1:500, AQP3- 1:500, and AQP5- 1:500). After washing with TBST, membranes were incubated with the appropriate secondary antibodies for 1 h at room temperature. The protein bands were visualized using the Miracle-Star™ Western blot detection system (iNtRON Biotechnology) and imaged using the ImageQuantTM LAS500 system (GE Healthcare Life Sciences, Issaquah, WA, USA). Densitometry data were obtained after Western blot analysis, and the band intensities were quantified using Amersham Imager 680 analysis software (GE Healthcare Life Sciences, Chicago, IL, USA) and normalized to β-actin as an internal control. For phosphorylated proteins, expression levels were normalized to their corresponding total protein levels (e.g., p-NF-κB/NF-κB, p-ERK/ERK, and p-p38/p38). For membrane stripping and reprobing, membranes were incubated in stripping buffer (iNtRON Biotechnology, Seongnam, Republic of Korea) at 37 °C for 20–25 min while protected from light, followed by three washes with TBST (10 min each). The membranes were then re-blocked for 1 h at room temperature and re-incubated with a different primary antibody, followed by incubation with the corresponding secondary antibody. Reprobed membranes were developed and quantified as described above. All experiments were performed at least three times independently. For proteins analyzed on the same PVDF membrane following stripping and reprobing, the corresponding β-actin band from that membrane was used as the common loading control. Proteins analyzed on independent membranes were normalized to the β-actin obtained from their respective membranes. To improve transparency, the membranes assigned for each Western blot panel are specified in the corresponding figure legends and Supplementary Figures. Uncropped original blot images are provided in the Supplementary Materials.

### 2.10. Measurement of Tear Secretion (Schirmer Tear Test)

Tear secretion was assessed using sterile Schirmer tear test strips (Kashsurg Schirmer Tear Test Strips; 5 mm × 35 mm), designed according to the standard Schirmer I test principle and equipped with millimeter scale markings for direct measurement of tear migration length. To minimize movement and stress during the procedure, rats were anesthetized using inhalational isoflurane (Ifran Solution, Seoul, Republic of Korea). After achieving adequate anesthesia, a Schirmer strip was gently inserted into the lower conjunctival fornix at approximately one-third of the lateral eyelid margin. The eyelids were allowed to close naturally, and strips were placed in both eyes simultaneously. The strips were maintained in position for 5 min. Following removal, the length of the moistened (blue-stained) portion of each strip was immediately measured in millimeters using the printed calibration scale. Tear secretion was recorded separately for the left and right eyes, and the mean value of both eyes for each animal was used for statistical analysis (*n* = 10 animals per group). Thus, the individual animal was considered the experimental unit. Tear secretion was evaluated at four experimental time points: Day 0 (baseline, prior to SCP administration), Day 5 (final day of SCP injection), Day 10 (one week after initiation of oral administration), and Day 16 (final day of oral administration). All measurements were performed at approximately the same time of day under consistent environmental conditions to minimize potential variability associated with circadian rhythm, humidity, and handling. The same isoflurane anesthesia protocol was applied uniformly to all experimental groups during Schirmer testing to minimize movement and ensure consistent measurement conditions.

### 2.11. Corneal Fluorescein Staining Analysis

Corneal epithelial damage was evaluated using sodium fluorescein staining. Briefly, 20 μL of 1% sodium fluorescein solution was dropped into the conjunctival sac of each rat eye. After 90 s, excess dye was gently removed, and the corneal staining was observed under a slit-lamp microscope with a cobalt blue excitation filter, and fluorescein-positive areas were visualized as green fluorescence (*n* = 3 per group). Representative images were captured for each eye under identical exposure settings. Fluorescein-positive areas were quantified in both eyes of each animal, and the mean value from the two eyes was used for statistical analysis. Three randomly selected animals per group were included in this analysis. The captured images were analyzed using ImageJ software version 1.54s (NIH, USA). For each image, the corneal region was manually defined as the region of interest (ROI). Images were converted to grayscale, and a uniform threshold was applied to identify fluorescein-positive areas. The stained area was quantified and expressed as the percentage of fluorescein-positive area relative to the total corneal area. All image acquisition and analysis procedures were performed under standardized conditions to ensure consistency.

### 2.12. Hematoxylin and Eosin (H&E) Staining for Corneal and Lacrimal Gland Histopathology

Corneal and lacrimal gland tissues (*n* = 3 per group) were fixed in 10% neutral buffered formalin, dehydrated, embedded in paraffin, and sectioned at a thickness of 4–5 μm. The sections were deparaffinized in xylene and rehydrated through a graded ethanol series, followed by washing in distilled water. Sections were stained with H&E to evaluate general histological architecture. The stained sections were dehydrated, cleared, mounted, and observed under a light microscope. Morphometric analysis of the lacrimal gland was performed using ImageJ version 1.54s (NIH, USA), where individual acini were manually outlined as ROI. Acinar cell density was calculated as the number of acinar cell nuclei per unit acinar area and expressed as nuclei/mm^2^, while the acinar area fraction (%) was determined as the ratio of total acinar area to total tissue area multiplied by 100. Corneal thickness was measured from H&E-stained sections using calibrated ImageJ analysis and expressed in micrometers (μm). Corneal sections were additionally evaluated for epithelial integrity, stromal organization, and corneal thickness.

### 2.13. Periodic Acid-Schiff (PAS) Staining and Evaluation of Conjunctiva and Lacrimal Gland

Conjunctival and lacrimal gland tissues (*n* = 3 per group) were fixed in 10% neutral buffered formalin, embedded in paraffin, and sectioned at 4–5 μm thickness. Sections were deparaffinized in xylene and rehydrated through a graded ethanol series. PAS staining was performed to visualize goblet cells and mucin-containing secretory material. Sections were counterstained with hematoxylin, dehydrated, cleared, and mounted. Goblet cells in the conjunctiva were identified based on PAS+ magenta staining and counted in randomly selected high-power fields. The average number of goblet cells per field was calculated for each sample and used for statistical analysis. PAS-stained lacrimal gland sections were qualitatively evaluated for secretory characteristics and mucin distribution.

### 2.14. Histopathological Evaluation of Ocular Surface and Lacrimal Gland

Histopathological evaluation of the ocular surface and lacrimal gland was performed using paraffin-embedded sections (4–5 μm) prepared as described above. Histological assessment was conducted using H&E- and PAS-stained sections. All histological assessments were performed using coded tissue samples, and the investigators performing image acquisition and quantitative analysis were not informed of the treatment group allocation. Assessments were conducted under a light microscope. Assessment focused on the acinar organization and inflammatory cell infiltration in the lacrimal gland, goblet cell abundance and epithelial structure in the conjunctiva, and epithelial thickness, surface integrity, and stromal alterations in the cornea. Representative images were selected from biological replicates included in the quantitative analysis and are representative of the histopathological findings observed within each experimental group.

### 2.15. Immunofluorescence Analysis of MMP9, CD68, and TLR4 Expression

Paraffin-embedded tissue sections (*n* = 3 per group) were deparaffinized and rehydrated as described above. Antigen retrieval was performed, and sections were blocked with normal serum to prevent nonspecific binding. The sections were then incubated overnight at 4 °C with primary antibodies against MMP9, CD68, and TLR4. After washing with PBS, appropriate fluorophore-conjugated secondary antibodies were applied, and nuclei were counterstained with DAPI. The primary antibodies used for immunofluorescence were commercial antibodies, and their catalog numbers and suppliers are provided in Section 2.1. The stained sections were mounted and examined under a fluorescence microscope. Images from all experimental groups were acquired using identical microscope settings, including objective magnification, exposure time, gain, and illumination intensity, to allow direct comparison among groups. Fluorescence intensity or positive staining area was quantified using ImageJ software using identical threshold and analysis parameters for all samples. Representative images were selected from biological replicates showing staining patterns consistent with the corresponding quantitative analysis. Image acquisition and quantitative analyses were performed using coded tissue samples without knowledge of the treatment group allocation.

### 2.16. Statistical Analysis

All data are presented as mean ± standard deviation (SD). The sample size was *n* = 3 for in vitro experiments. For the in vivo experiments, the sample size varied according to the experimental endpoint and tissue allocation: *n* = 10 for body weight and tear secretion; *n* = 3 for corneal fluorescein staining, histological analyses (H&E, PAS, and immunofluorescence), and RT-PCR; and *n* = 6 for Western blot analysis. Statistical analyses were performed using GraphPad Prism 10.0.0 (GraphPad Software, San Diego, CA, USA). One-way ANOVA followed by Tukey’s post hoc multiple-comparison test was applied for single-endpoint analyses. Two-way repeated-measures ANOVA was used for analyses involving repeated measurements over time, including body weight and tear secretion. For ocular functional assessments, measurements from both eyes were averaged, and each animal was considered the experimental unit. A *p*-value < 0.05 was considered statistically significant. No animals or samples were excluded from the statistical analyses.

## 3. Results

### 3.1. Lute-Gen^®^ Protects HCE-T Cells from TNF-α-Induced Cytotoxicity

**Figure 2:**
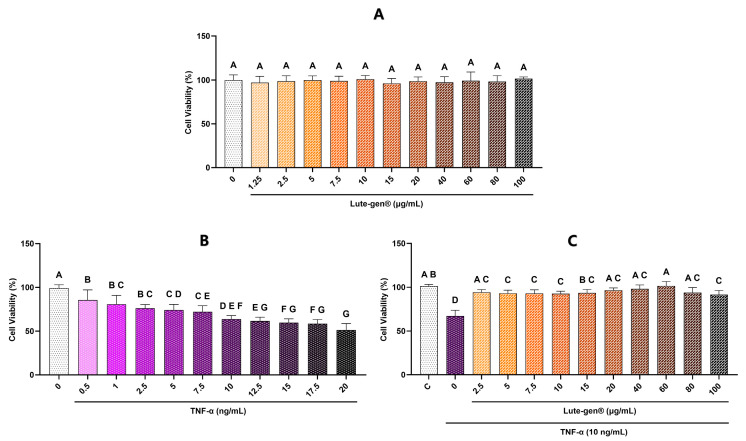
Effect of Lute-gen^®^ and TNF-α on HCE-T cell viability. Cell viability following treatment with (**A**) Lute-gen^®^ (1.25–100 μg/mL), (**B**) TNF-α (0.5–20 ng/mL), or (**C**) co-treatment with TNF-α (10 ng/mL) and Lute-gen^®^ (2.5–100 μg/mL). Data are presented as mean ± SD (*n* = 3). C, DMSO; TNF-α, tumor necrosis factor-alpha; and Lute-gen^®^, Lutein 20% (Free) with Zeaxanthin 4% Oil. Statistical significance is represented using letter annotations. Groups with different letters are significantly different (*p* < 0.05), whereas groups sharing at least one letter are not significantly different according to Tukey’s multiple comparisons test.

To evaluate the effect of Lute-gen^®^ on cell viability, HCE-T cells were treated with 1.25–100 μg/mL of Lute-gen^®^ for 24 h. Lute-gen^®^ did not exhibit significant cytotoxicity at any tested concentration (Figure 2A). To establish an inflammatory injury model, HCE-T cells were exposed to 0.5–20 ng/mL of TNF-α for 24 h. TNF-α reduced cell viability in a dose-dependent manner, and 10 ng/mL was selected for subsequent experiments (Figure 2B). Co-treatment with Lute-gen^®^ (2.5–100 μg/mL) significantly improved the TNF-α-induced reduction in cell viability (Figure 2C). As comparable protective effects were observed across the tested concentrations, 2.5, 5, and 7.5 μg/mL were selected for subsequent experiments.

### 3.2. Lute-Gen^®^ Enhances Antioxidant Defense System and Attenuates ROS Accumulation in TNF-α-Stimulated HCE-T Cells

**Figure 3:**
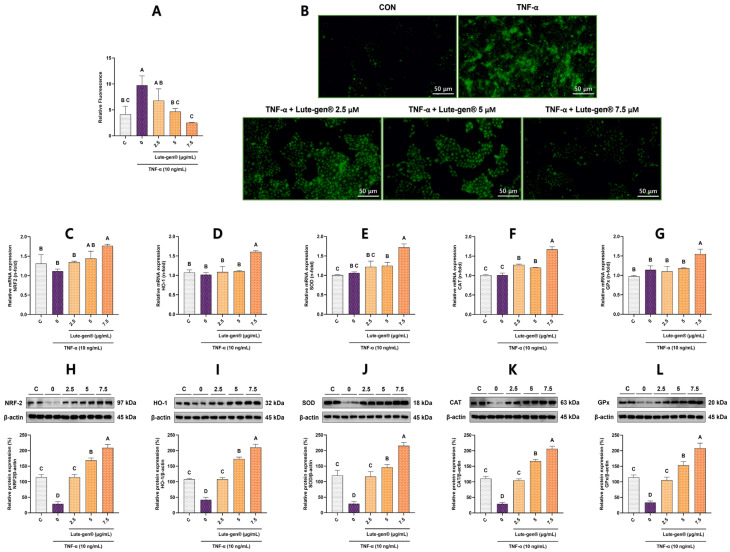
Effect of Lute-gen^®^ on antioxidant gene and protein expression in TNF-α-stimulated HCE-T cells. (**A**,**B**) Representative images and quantification of intracellular ROS levels. (**C**–**G**) Relative mRNA and (**H**–**L**) protein expression levels of Nrf2, HO-1, SOD, CAT, and GPx. Data are presented as mean ± SD (*n* = 3 biological replicates). Panels (**H**–**L**) were analyzed on independent membranes; therefore, each target protein was normalized to its corresponding β-actin loading control obtained from the same membrane. Uncropped original blots are shown in Supplementary Figure S1. C, control; TNF-α, tumor necrosis factor-alpha; Lute-gen^®^, Lutein 20% (Free) with Zeaxanthin 4% Oil; ROS, reactive oxygen species; Nrf2, nuclear factor erythroid 2-related factor 2; HO-1, heme oxygenase-1; SOD, superoxide dismutase; CAT, catalase; and GPx, glutathione peroxidase. Statistical significance is represented using letter annotations. Groups with different letters are significantly different (*p* < 0.05), whereas groups sharing at least one letter are not significantly different according to Tukey’s multiple comparisons test.

To evaluate the effect of Lute-gen^®^ on intracellular OS, the ROS levels were assessed using DCFH-DA staining. TNF-α stimulation markedly increased intracellular ROS generation compared with the control group, whereas Lute-gen^®^ reduced intracellular ROS accumulation, with the greatest reduction observed at 7.5 μg/mL. Representative fluorescence images were consistent with the quantitative analysis (Figure 3A,B). The mRNA and protein expression levels of Nrf2, HO-1, SOD, CAT, and GPx were subsequently evaluated. TNF-α did not alter the mRNA expression levels of Nrf2, HO-1, SOD, CAT, and GPx compared with the control group, whereas the corresponding protein expression levels were significantly reduced. Lute-gen^®^ increased the mRNA expression of all antioxidant genes, with the highest expression levels generally observed at 7.5 μg/mL (Figure 3C–G), while significantly restoring TNF-α-induced reduction in the corresponding protein expression levels (Figure 3H–L). All full-length blots are provided in the Supplementary Material section for transparency and data integrity (Supplementary Figure S1).

### 3.3. Lute-Gen^®^ Suppresses TNF-α-Induced TLR4/MyD88/NF-κB/NLRP3 Inflammasome Signaling in HCE-T Cells

**Figure 4:**
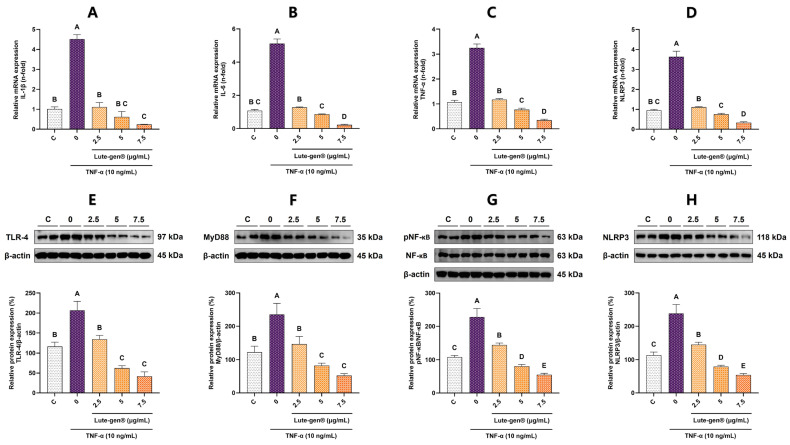
Effect of Lute-gen^®^ on inflammatory gene and protein expression in TNF-α-stimulated HCE-T cells. (**A**–**D**) Relative mRNA expression levels of IL-1β, IL-6, TNF-α, and NLRP3. (**E**–**H**) Protein expression levels of TLR4, MyD88, p-NF-κB/NF-κB, and NLRP3. Data are presented as mean ± SD (*n* = 3 biological replicates). Panels (**E**,**F**) (TLR4 and MyD88) were sequentially detected on the same membrane following stripping and reprobing and therefore share the same β-actin loading control. Panels (**G**,**H**) were analyzed on independent membranes with their respective loading controls. Uncropped original blots are shown in Supplementary Figure S2. C, control; TNF-α, tumor necrosis factor-alpha; Lute-gen^®^, Lutein 20% (Free) with Zeaxanthin 4% Oil; IL, interleukin; NLRP3, NOD-like receptor family pyrin domain-containing 3; TLR4, Toll-like receptor 4; MyD88, myeloid differentiation primary response 88; p, phosphorylated; and NF-κB, nuclear factor kappa B. Statistical significance is represented using letter annotations. Groups with different letters are significantly different (*p* < 0.05), whereas groups sharing at least one letter are not significantly different according to Tukey’s multiple comparisons test.

To assess the anti-inflammatory effects of Lute-gen^®^, the mRNA expression levels of IL-1β, IL-6, TNF-α, and NLRP3, together with the protein expression levels of TLR4, MyD88, p-NF-κB/NF-κB, and NLRP3, were evaluated. TNF-α stimulation significantly increased the mRNA expression of IL-1β, IL-6, TNF-α, and NLRP3 compared with the control group. Lute-gen^®^ significantly reduced the mRNA expression of these inflammatory genes, with the lowest expression levels generally observed at 7.5 μg/mL (Figure 4A–D). At the protein level, TNF-α significantly increased the expression of TLR4, MyD88, p-NF-κB/NF-κB, and NLRP3, whereas Lute-gen^®^ significantly attenuated this increase across all tested concentrations (Figure 4E–H). Overall, Lute-gen^®^ treatment was associated with reduced expression of inflammatory cytokines and inflammatory signaling-related proteins in TNF-α-stimulated HCE-T cells. All full-length blots are provided in the Supplementary Material section for transparency and data integrity (Supplementary Figure S2).

### 3.4. Lute-Gen^®^ Restores Aquaporin Expression in TNF-α-Stimulated HCE-T Cells

**Figure 5:**
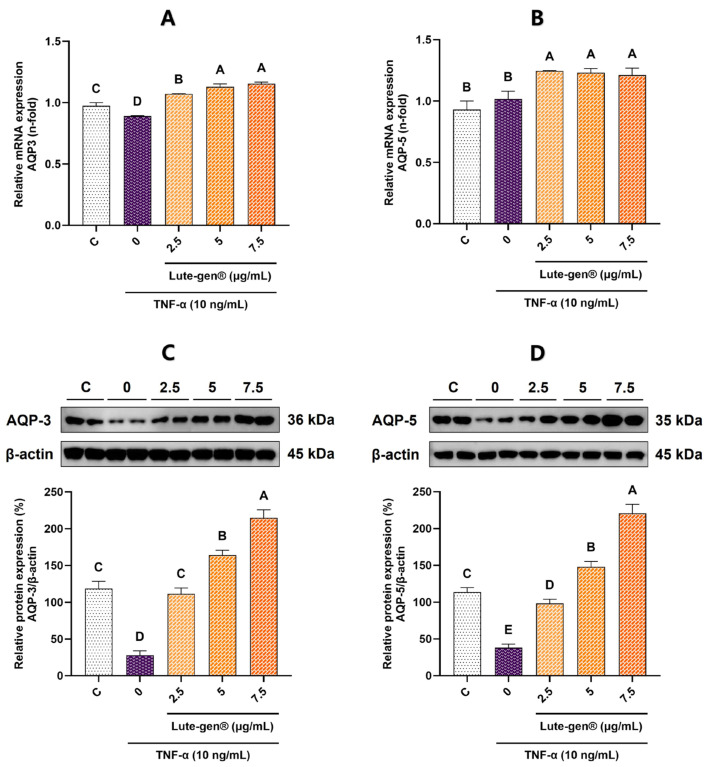
Effect of Lute-gen^®^ on aquaporin expression in TNF-α-stimulated HCE-T cells. (**A**,**B**) Relative mRNA and (**C**,**D**) protein expression levels of AQP3 and AQP5. Data are presented as mean ± SD (*n* = 3 biological replicates). Panels (**C**,**D**) were analyzed on independent membranes and normalized to their corresponding β-actin loading controls. Uncropped original blots are shown in Supplementary Figure S3. D, DMSO; TNF-α, tumor necrosis factor-alpha; Lute-gen^®^, Lutein 20% (Free) with Zeaxanthin 4% Oil; and AQP, aquaporin. Statistical significance is represented using letter annotations. Groups with different letters are significantly different (*p* < 0.05), whereas groups sharing at least one letter are not significantly different according to Tukey’s multiple comparisons test.

To evaluate the effect of Lute-gen^®^ on aquaporin expression, the mRNA and protein expression levels of AQP3 and AQP5 were analyzed. TNF-α significantly reduced the mRNA expression of AQP3, whereas AQP5 mRNA remained comparable to that of the control group. However, the corresponding protein expression levels of both AQP3 and AQP5 were significantly reduced. Lute-gen^®^ increased the mRNA expression of both AQP3 and AQP5 and significantly restored the TNF-α-induced reduction in the corresponding protein expression levels (Figure 5A–D). Overall, Lute-gen^®^ treatment was associated with restoration of aquaporin expression at both the mRNA and protein levels in TNF-α-stimulated HCE-T cells. All full-length blots are provided in the Supplementary Material section for transparency and data integrity (Supplementary Figure S3).

### 3.5. Lute-Gen^®^ Restores Tear Production in SCP-Induced Dry Eye Rats Without Affecting Body Weight

**Figure 6:**
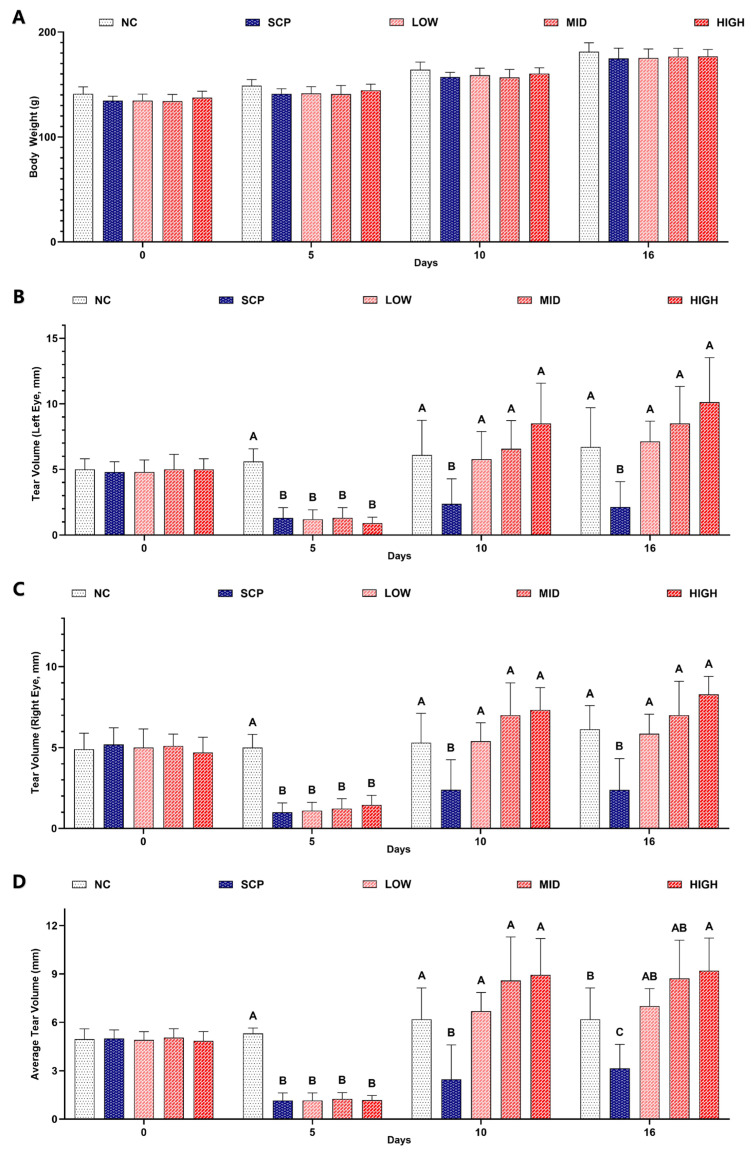
Effect of Lute-gen^®^ on body weight and tear production in SCP-induced dry eye rats. (**A**) Body weight changes during the experimental period. (**B**–**D**) Tear secretion measured on Days 0, 5, 10, and 16 in the left eye, right eye, and as the average of both eyes. Data are presented as mean ± SD (*n* = 10 animals per group). NC, normal control; SCP, scopolamine-treated group; LOW, Lute-gen^®^ (1 mg/kg); MID, Lute-gen^®^ (5 mg/kg); and HIGH, Lute-gen^®^ (10 mg/kg). Statistical significance is represented using letter annotations. Groups with different letters are significantly different (*p* < 0.05), whereas groups sharing at least one letter are not significantly different according to Tukey’s multiple comparisons test.

To evaluate whether Lute-gen^®^ affected general growth, body weight was monitored throughout the experimental period. Body weight increased steadily in all groups, with no significant differences observed among the groups (Figure 6A). These findings indicate that SCP administration and oral treatment with Lute-gen^®^ were not associated with any systemic toxicity or adverse effects on general growth. Consistently, no abnormal behavior or obvious welfare loss was observed during the study period, and food and water intake remained comparable among the groups (Supplementary Figure S4). Following completion of SCP induction (Day 5), all SCP-administered groups exhibited significantly reduced tear secretion compared with the NC group, confirming successful induction of dry eye before the assessment of treatment effect. Following oral administration of Lute-gen^®^, tear secretion increased by Day 10 and was maintained through Day 16 in all treatment groups compared with the SCP group (Figure 6B–D). Overall, Lute-gen^®^ treatment was associated with improved tear secretion in SCP-induced dry eye rats without affecting body weight.

### 3.6. Lute-Gen^®^ Attenuates Corneal Epithelial Damage in SCP-Induced Dry Eye Rats

**Figure 7:**
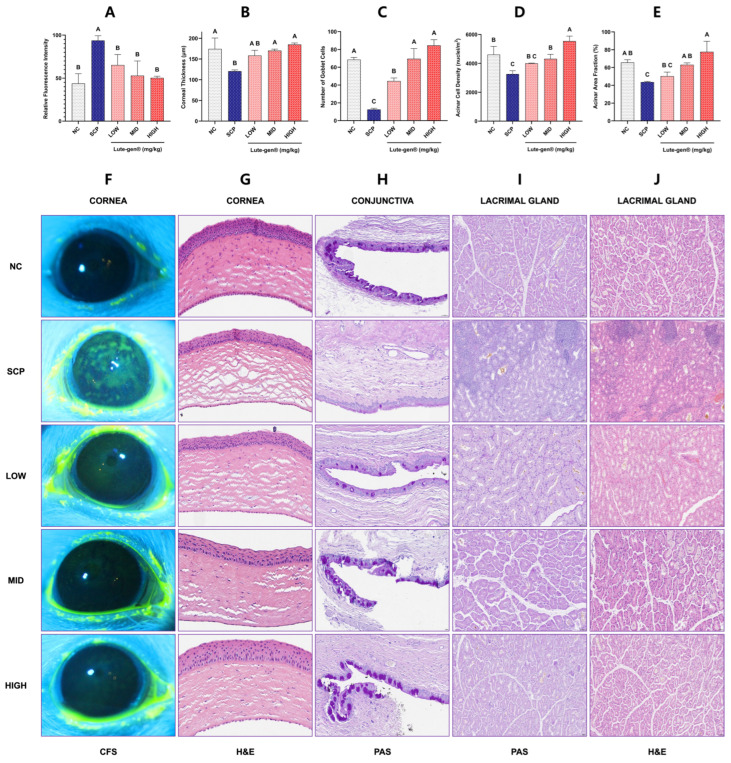
Effect of Lute-gen^®^ on corneal epithelial damage and histopathological changes in the conjunctiva, cornea, and lacrimal gland of SCP-induced dry eye rats. Quantification of (**A**) corneal fluorescein staining intensity, (**B**) corneal thickness, (**C**) PAS-positive goblet cells in conjunctival tissues, (**D**) acinar cell density in lacrimal gland tissues, and (**E**) acinar area fraction in lacrimal gland tissues. (**F**–**J**) Representative corneal fluorescein and histological images of the cornea (H&E), conjunctiva (PAS), and lacrimal gland (PAS and H&E). Data are presented as mean ± SD (*n* = 3 animals per group). Scale bar = 50 μm, magnification 100×. NC, normal control; SCP, scopolamine-treated group; LOW, Lute-gen^®^ (1 mg/kg); MID, Lute-gen^®^ (5 mg/kg); HIGH, Lute-gen^®^ (10 mg/kg); PAS, Periodic Acid-Schiff; H&E, Hematoxylin and Eosin; and CFS, corneal fluorescein staining. Statistical significance is represented using letter annotations. Groups with different letters are significantly different (*p* < 0.05), whereas groups sharing at least one letter are not significantly different according to Tukey’s multiple comparisons test.

Corneal fluorescein staining was performed to evaluate corneal epithelial damage following SCP-induced dry eye. Compared with the NC group, the SCP group exhibited markedly increased fluorescein staining, indicating successful induction of ocular surface damage. Representative images showed reduced corneal fluorescein staining following Lute-gen^®^ treatment compared with the SCP group (Figure 7F). Quantitative analysis supported these observations (Figure 7A). The SCP group showed significantly increased fluorescence intensity compared with the NC group, whereas Lute-gen^®^ significantly reduced fluorescence intensity at all tested doses. No significant differences were observed among the LOW, MID, and HIGH treatment groups. Overall, Lute-gen^®^ treatment was associated with reduced corneal fluorescein staining in SCP-induced dry eye rats.

### 3.7. Lute-Gen^®^ Attenuates Corneal Epithelial Damage and Restores Corneal Thickness in SCP-Induced Dry Eye Rats

H&E staining of corneal sections revealed structural alterations following SCP induction. In the NC group, the corneal epithelium appeared well-organized with preserved epithelial continuity and normal thickness. In contrast, the SCP group exhibited epithelial thinning, reduced epithelial stratification, and surface irregularity, consistent with ocular surface damage associated with dry eye. Representative H&E images showed that Lute-gen^®^ treatment improved epithelial continuity, reduced surface irregularity, and partially restored epithelial thickness and overall tissue architecture compared with the SCP group (Figure 7G). Quantitative analysis confirmed that corneal thickness was significantly reduced in the SCP group compared with the NC group and was significantly restored in the MID and HIGH groups following Lute-gen^®^ treatment (Figure 7B). The LOW group showed partial recovery but did not differ significantly from either the NC or SCP group. Overall, Lute-gen^®^ treatment was associated with improved corneal morphology and restoration of corneal thickness in SCP-induced dry eye rats.

### 3.8. Lute-Gen^®^ Restores Conjunctival Goblet Cells and Mucin Production in SCP-Induced Dry Eye Rats

PAS staining of conjunctival tissues revealed marked alterations in goblet cell distribution and mucin content following SCP induction. In the NC group, the conjunctival epithelium exhibited abundant PAS-positive goblet cells with strong magenta staining. In contrast, the SCP group showed a marked reduction in PAS-positive goblet cells, accompanied by diminished mucin staining and epithelial irregularity. Representative PAS images showed that Lute-gen^®^ treatment increased PAS-positive goblet cell abundance, enhanced mucin staining, and improved epithelial morphology compared with the SCP group (Figure 7H). Quantitative analysis confirmed that goblet cell numbers were significantly reduced in the SCP group compared with the NC group and were significantly increased following Lute-gen^®^ treatment, with goblet cell numbers in the MID and HIGH groups reaching levels comparable to those of the NC group (Figure 7C). Overall, Lute-gen^®^ treatment was associated with restoration of conjunctival goblet cells and mucin staining in SCP-induced dry eye rats.

### 3.9. Lute-Gen^®^ Preserves Lacrimal Gland Architecture and Reduces Inflammatory Infiltration in SCP-Induced Dry Eye Rats

Histological evaluation of the lacrimal gland using PAS and H&E staining revealed marked structural alterations following SCP administration. In the NC group, the lacrimal gland exhibited well-organized acinar units with uniform morphology and minimal interstitial space. In contrast, the SCP group showed marked acinar disorganization, reduced acinar density, expansion of interstitial regions, and inflammatory cell infiltration. PAS staining also demonstrated reduced secretory material and diminished acinar integrity compared with the NC group. Representative PAS and H&E images showed that Lute-gen^®^ treatment improved acinar organization, reduced inflammatory cell infiltration, and preserved overall glandular morphology compared with the SCP group (Figure 7I,J). Quantitative analysis confirmed that acinar cell density and acinar area fraction were significantly reduced in the SCP group compared with the NC group and were significantly increased following Lute-gen^®^ treatment, with the MID and HIGH groups exhibiting values comparable to those of the NC group (Figure 7D,E). Overall, Lute-gen^®^ treatment was associated with preservation of lacrimal gland architecture and reduced histopathological alterations in SCP-induced dry eye rats.

### 3.10. Lute-Gen^®^ Enhances Antioxidant Defense System in SCP-Induced Dry Eye Rats

**Figure 8:**
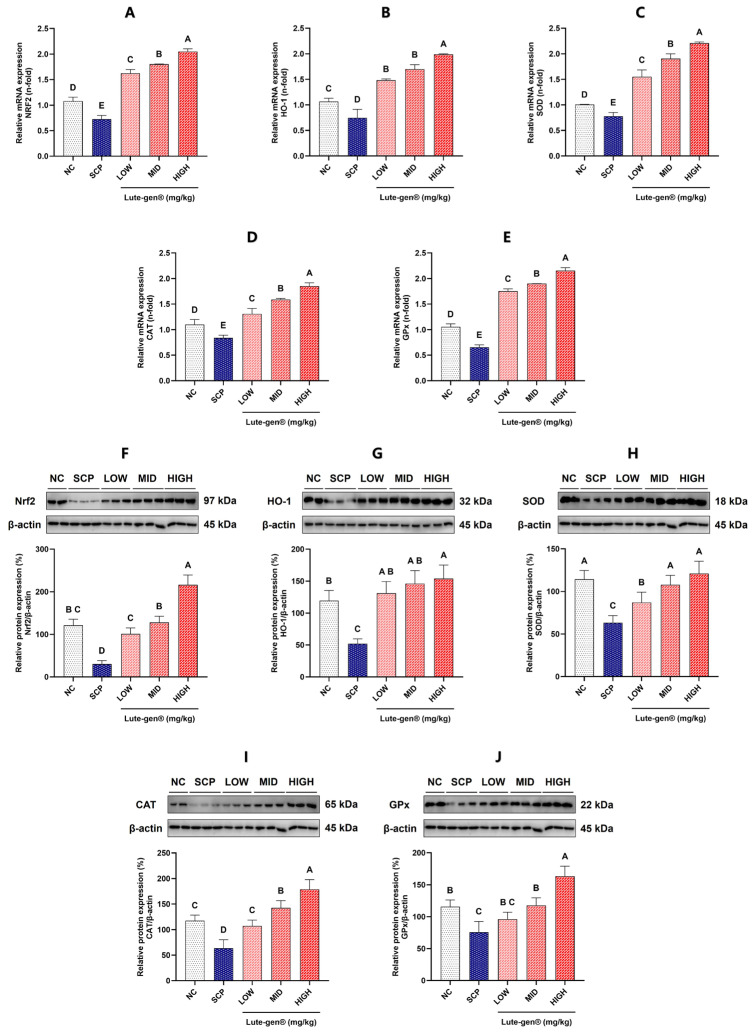
Effect of Lute-gen^®^ on antioxidant gene and protein expression in SCP-induced dry eye rats. (**A**–**E**) Relative mRNA and (**F**–**J**) protein expression levels of Nrf2, HO-1, SOD, CAT, and GPx in corneal tissues. Data are presented as mean ± SD (mRNA, *n* = 3 animals per group; protein, *n* = 6 animals per group). Panels (**F**,**H**–**J**) (Nrf2, SOD, CAT, and GPx) were sequentially detected on the same membrane following stripping and reprobing and therefore share the same β-actin loading control. Panel (**G**) (HO-1) was analyzed on an independent membrane with its corresponding β-actin loading control. Uncropped original blots are shown in Supplementary Figure S5. NC, normal control; SCP, scopolamine-treated group; LOW, Lute-gen^®^ (1 mg/kg); MID, Lute-gen^®^ (5 mg/kg); HIGH, Lute-gen^®^ (10 mg/kg); Nrf2, nuclear factor erythroid 2-related factor 2; HO-1, heme oxygenase-1; SOD, superoxide dismutase; CAT, catalase; and GPx, glutathione peroxidase. Statistical significance is represented using letter annotations. Groups with different letters are significantly different (*p* < 0.05), whereas groups sharing at least one letter are not significantly different according to Tukey’s multiple comparisons test.

The mRNA and protein expression levels of Nrf2, HO-1, SOD, CAT, and GPx were evaluated in corneal tissues. SCP administration significantly reduced the mRNA and protein expression levels of these antioxidant markers compared with the NC group. Lute-gen^®^ significantly increased the mRNA expression of Nrf2, HO-1, SOD, CAT, and GPx (Figure 8A–E) and significantly restored the SCP-induced reduction in their protein expression (Figure 8F–J). The HIGH group generally exhibited the highest expression levels, although differences among the Lute-gen^®^ treatment groups were not consistently significant for all markers. Overall, Lute-gen^®^ treatment was associated with increased antioxidant marker expression at both the transcriptional and translational levels in SCP-induced dry eye rats. All full-length blots are provided in the Supplementary Material section for transparency and data integrity (Supplementary Figure S5).

### 3.11. Lute-Gen^®^ Suppresses Inflammatory Gene Expression and Downregulates TLR4/NF-κB-Inflammasome and MAPK/MMP Signaling in SCP-Induced Dry Eye Rats

**Figure 9:**
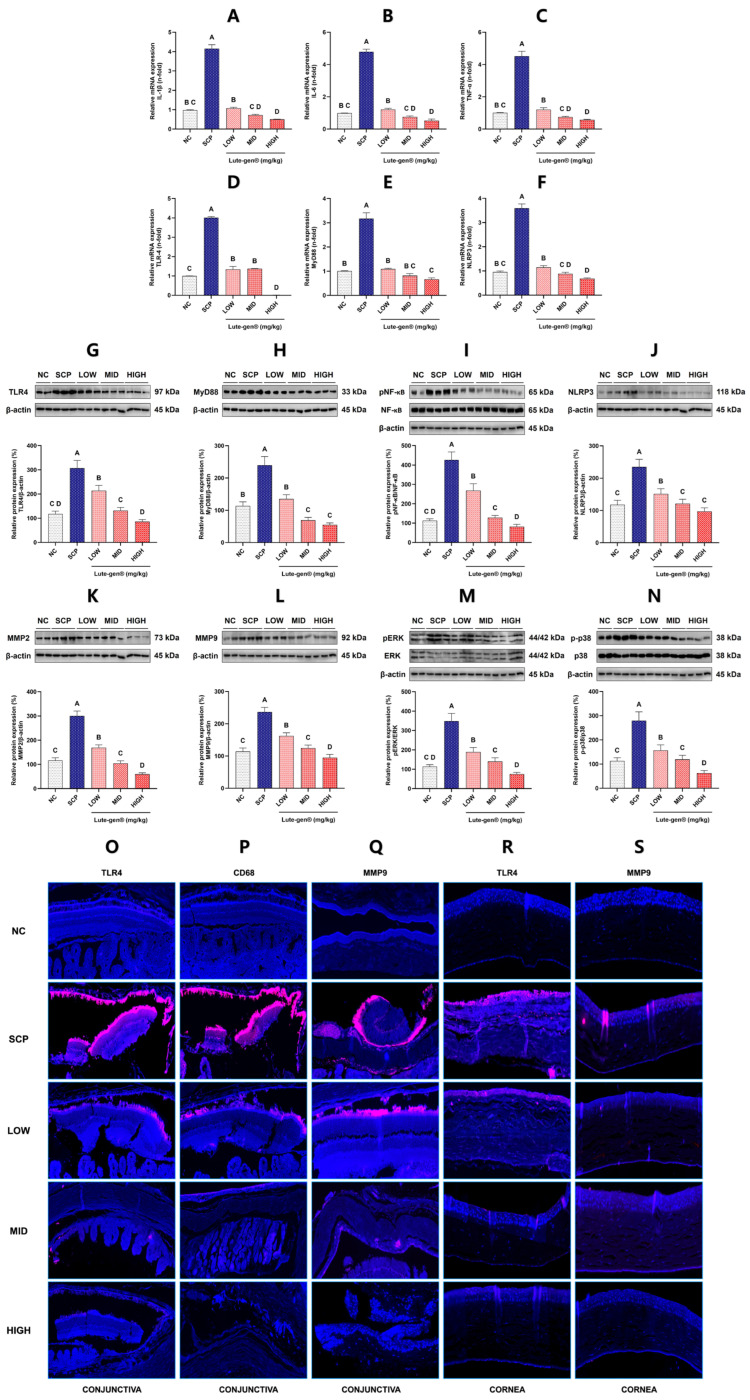
Effect of Lute-gen^®^ on inflammatory signaling and tissue injury-related protein expression in SCP-induced dry eye rats. (**A**–**F**) Relative mRNA expression levels of IL-1β, IL-6, TNF-α, TLR4, MyD88 and NLRP3 in corneal tissues. (**G**–**N**) Protein expression levels of TLR4, MyD88, p-NF-κB/NF-κB, NLRP3, MMP2, MMP9, p-ERK/ERK, and p-p38/p38 in corneal tissues. Data are presented as mean ± SD (mRNA, *n* = 3 animals per group; protein, *n* = 6 animals per group). Panels (**G**,**J**,**K**) (TLR4, NLRP3, and MMP2) were sequentially detected on the same membrane following stripping and reprobing and therefore share the same β-actin loading control. Panel (**H**) (MyD88) was analyzed on an independent membrane with its corresponding β-actin loading control. Panels (**I**,**M**,**N**) represent phosphorylated/total protein pairs (p-NF-κB/NF-κB, p-ERK/ERK, and p-p38/p38) and L (MMP9), each detected sequentially on the same membrane and normalized to their respective total protein. Uncropped original blots are shown in Supplementary Figure S6. (**O**–**Q**) Representative immunofluorescence images of TLR4, CD68, and MMP9 in conjunctival tissues and (**R**,**S**) TLR4 and MMP9 in corneal tissues. Scale bar = 100 μm, magnification 200×. Immunofluorescence images are representative of *n* = 3 animals per group. NC, normal control; SCP, scopolamine-treated group; LOW, Lute-gen^®^ (1 mg/kg); MID, Lute-gen^®^ (5 mg/kg); HIGH, Lute-gen^®^ (10 mg/kg); IL, interleukin; NLRP3, NOD-like receptor family pyrin domain containing 3; TLR4, toll-like receptor 4; MyD88, myeloid differentiation primary response protein 88; p-, phosphorylated; NF-κB, nuclear factor kappa light chain enhancer of activated B cells; MMP, matrix metalloproteinase; ERK, extracellular signal-regulated kinase; p38, p38 mitogen-activated protein kinase; and CD, cluster of differentiation. Statistical significance is represented using letter annotations. Groups with different letters are significantly different (*p* < 0.05), whereas groups sharing at least one letter are not significantly different according to Tukey’s multiple comparisons test.

The mRNA expression levels of IL-1β, IL-6, TNF-α, TLR4, MyD88, and NLRP3, together with the protein expression levels of TLR4, MyD88, p-NF-κB/NF-κB, NLRP3, MMP2, MMP9, p-ERK/ERK, and p-p38/p38, were evaluated in corneal tissues. SCP administration significantly increased the mRNA and protein expression levels of these inflammatory markers compared with the NC group. Lute-gen^®^ significantly reduced the mRNA expression of IL-1β, IL-6, TNF-α, TLR4, MyD88, and NLRP3 (Figure 9A–F) and significantly attenuated the SCP-induced increase in TLR4, MyD88, p-NF-κB/NF-κB, NLRP3, MMP2, MMP9, p-ERK/ERK, and p-p38/p38 protein expression (Figure 9G–N). Overall, Lute-gen^®^ treatment was associated with reduced expression of inflammatory cytokines, inflammatory signaling-related proteins, and tissue injury-associated markers in SCP-induced dry eye rats. All full-length blots are provided in the Supplementary Material section for transparency and data integrity (Supplementary Figure S6).

### 3.12. Lute-Gen^®^ Suppresses TLR4, CD68, and MMP9-Associated Inflammatory Responses in Conjunctival and Corneal Tissues of SCP-Induced Dry Eye Rats

Immunofluorescence staining was performed to evaluate TLR4, CD68, and MMP9 expression in conjunctival and corneal tissues following SCP administration. In the conjunctiva, the SCP group showed stronger TLR4 and MMP9 fluorescence signals together with increased CD68-positive staining compared with the NC group. Representative immunofluorescence images showed reduced TLR4, CD68, and MMP9 fluorescence staining following Lute-gen^®^ treatment compared with the SCP group (Figure 9O–Q). Similarly, SCP administration increased TLR4 and MMP9 fluorescence staining in the cornea, whereas Lute-gen^®^ treatment reduced the fluorescence signals of both markers (Figure 9R,S). Different-magnification views of the conjunctival and corneal immunofluorescence images are provided in Supplementary Figures S7–S11. Representative images were acquired using identical microscope settings for all experimental groups. Overall, Lute-gen^®^ treatment was associated with reduced TLR4, CD68, and MMP9 immunofluorescence staining in conjunctival and corneal tissues of SCP-induced dry eye rats.

### 3.13. Lute-Gen^®^ Restores Aquaporin Expression in SCP-Induced Dry Eye Rats

**Figure 10:**
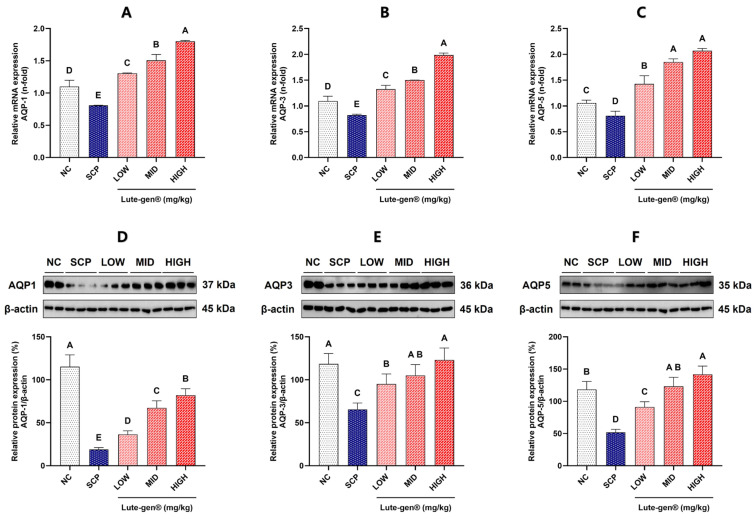
Effect of Lute-gen^®^ on aquaporin gene and protein expression in SCP-induced dry eye rats. (**A**–**C**) Relative mRNA and (**D**–**F**) protein expression levels of AQP1, AQP3, and AQP5 in corneal tissues. Data are presented as mean ± SD (mRNA, *n* = 3 animals per group; protein, *n* = 6 animals per group). Panels (**D**,**F**) (AQP1 and AQP5) were sequentially detected on the same membrane following stripping and reprobing and therefore share the same β-actin loading control. Panel (**E**) (AQP3) was analyzed on an independent membrane with its corresponding β-actin loading control. Uncropped original blots are shown in Supplementary Figure S12. NC, normal control; SCP, scopolamine-treated group; LOW, Lute-gen^®^ (1 mg/kg); MID, Lute-gen^®^ (5 mg/kg); HIGH, Lute-gen^®^ (10 mg/kg); and AQP, aquaporin. Statistical significance is represented using letter annotations. Groups with different letters are significantly different (*p* < 0.05), whereas groups sharing at least one letter are not significantly different according to Tukey’s multiple comparisons test.

The mRNA and protein expression levels of AQP1, AQP3, and AQP5 were evaluated in corneal tissues. SCP administration significantly reduced the mRNA and protein expression levels of all three aquaporins compared with the NC group. Lute-gen^®^ significantly increased the mRNA expression of AQP1, AQP3, and AQP5 (Figure 10A–C) and significantly restored the SCP-induced reduction in their protein expression (Figure 10D–F). The HIGH group generally exhibited the highest expression levels, although differences among the Lute-gen^®^ treatment groups were not consistently significant for all aquaporins. Overall, Lute-gen^®^ treatment was associated with restoration of aquaporin expression at both the transcriptional and translational levels in SCP-induced dry eye rats. All full-length blots are provided in the Supplementary Material section for transparency and data integrity (Supplementary Figure S12).

## 4. Discussion

DED is a multifactorial ocular surface disorder characterized by OS, inflammation, and disruption of tear film homeostasis. Histopathological alterations include corneal and conjunctival epithelial damage, loss of mucin-secreting goblet cells, and meibomian gland dysfunction, all of which contribute to tear film instability [34]. Given its high global prevalence, there is a critical need for therapeutic strategies that can simultaneously target multiple pathological pathways.

TNF-α has been widely used to mimic inflammatory conditions associated with DED in vitro [35]. In the present study, an in vitro DED model was established using TNF-α stimulation in HCE-T cells, effectively recapitulating key pathological features of DED, including increased pro-inflammatory cytokine production, OS, and disruption of epithelial homeostasis [5,36,37]. Dose optimization experiments identified 10 ng/mL TNF-α as the optimal concentration to induce significant cellular injury in HCE-T cells, thereby providing a suitable in vitro inflammatory model for subsequent experiments. Furthermore, Lute-gen^®^ exhibited no cytotoxicity across the tested range and demonstrated significant cytoprotective effects. Based on these findings, concentrations of 2.5, 5, and 7.5 μg/mL were selected for subsequent experiments to evaluate their protective effects against TNF-α-induced cellular damage. Although hyperosmolar stress is recognized as a primary initiating factor in DED and is widely used in experimental models [1,4], TNF-α stimulation represents a well-established in vitro model for investigating downstream inflammatory and oxidative responses associated with ocular surface injury [5]. Because the primary objective of the present in vitro experiments was to evaluate the effects of Lute-gen^®^ on inflammatory signaling and antioxidant responses, TNF-α stimulation was selected as an appropriate and reproducible model. Nevertheless, TNF-α stimulation does not fully recapitulate the complex pathophysiology of DED, and future studies employing hyperosmolar stress or combined experimental models will be valuable for further evaluating the protective effects of Lute-gen^®^.

TLR4, expressed on the surface of corneal epithelial cells, plays a pivotal role in initiating innate immune responses under DED-associated stress conditions. Upon ligand binding, TLR4 recruits the adaptor protein MyD88, leading to the formation of a downstream signaling complex that activates the NF-κB signaling pathway via degradation of its inhibitory protein IκB. This activation results in the nuclear translocation of NF-κB and subsequent transcription of pro-inflammatory mediators, such as IL-1β, TNF-α, and IL-6, as well as NLRP3. Collectively, this signaling cascade amplifies ocular surface inflammation and contributes to the progression of DED pathology [38]. In the present study, TNF-α stimulation was associated with the activation of the TLR4/MyD88/NF-κB signaling pathway axis in HCE-T cells, as evidenced by the upregulation of transcriptional levels of TLR4, MyD88, and NLRP3, along with increased protein expression of TLR4, MyD88, p-NF-κB/NF-κB, and NLRP3. These findings indicate the establishment of an inflammatory environment characteristic of DED. Previous studies have reported that *Aster koraiensis* extract reduces pro-inflammatory cytokine production, including IL-1β, TNF-α, and IL-6, in TNF-α-induced ARPE-19 cells [39]. Consistent with these observations, Lute-gen^®^ treatment was associated with reduced transcriptional expression of inflammatory cytokines together with decreased protein expression of TLR4, MyD88, p-NF-κB/NF-κB, and NLRP3 in TNF-α-stimulated HCE-T cells. Moreover, TNF-α-induced mRNA expression levels of pro-inflammatory cytokines, including IL-1β, TNF-α, and IL-6, were markedly reduced following Lute-gen^®^ treatment, further supporting its anti-inflammatory potential in this experimental model.

OS is now recognized as a central component of dry eye pathophysiology, and TFOS DEWS II identified oxidative injury as an important part of the vicious cycle that perpetuates ocular surface disease [4,10,11]. In parallel, NFE2L2/Nrf2-related antioxidant responses have been shown to protect corneal epithelial cells and ameliorate dry eye changes in various experimental settings [13,14,40]. In the present study, TNF-α stimulation markedly increased intracellular ROS levels in HCE-T cells, accompanied by a compromised antioxidant defense system, as evidenced by reduced protein expression of Nrf2, HO-1, CAT, GPx, and SOD. These findings are consistent with the establishment of an oxidative stress-associated cellular environment characteristic of DED. A recent study using LPS-induced human corneal epithelial HCEC and RAW 264.7 cells demonstrated that gallic acid effectively reduced intracellular ROS through activation of the Nrf2/HO-1 axis [41]. Consistent with these findings, Lute-gen^®^ reduced intracellular ROS accumulation and was associated with increased mRNA and protein expression of Nrf2, HO-1, CAT, GPx, and SOD. Collectively, these findings suggest that the protective effects of Lute-gen^®^ may be associated with modulation of antioxidant defense pathways, including the Nrf2/HO-1 axis, thereby contributing to enhanced cellular antioxidant capacity and reduced TNF-α-induced oxidative stress.

Aquaporins are important for transmembrane water movement in the ocular surface and lacrimal tissues, and altered AQP expression has been associated with tear secretion abnormalities and dry eye-related gland dysfunction [23,42,43]. In the present study, TNF-α stimulation led to a marked downregulation of both mRNA and protein expression levels of AQP3 and AQP5 in HCE-T cells, reflecting impaired water transport capacity under inflammatory stress. Notably, Lute-gen^®^ significantly restored the expression of AQP3 and AQP5 at both transcriptional and translational levels across all tested concentrations, suggesting that restoration of aquaporin expression may contribute to the beneficial effects of Lute-gen^®^ on ocular surface hydration.

Systemic SCP administration is a well-established model of aqueous-deficient dry eye that reduces tear secretion and induces ocular surface and lacrimal gland alterations relevant to human disease [44,45,46,47]. This model has been widely used to investigate key pathogenic mechanisms of DED, including inflammation, oxidative stress, tear film instability, and epithelial damage. SCP-induced cholinergic blockade impairs lacrimal gland function, resulting in reduced aqueous tear production and subsequent ocular surface desiccation, which promotes inflammation, oxidative stress, goblet cell loss, corneal epithelial injury, and disruption of tear film homeostasis. The present study employed the SCP-induced dry eye model in SD rats to investigate the protective effects of Lute-gen^®^ on functional, histopathological, and molecular changes associated with experimental DED.

To evaluate the effects of different oral doses of Lute-gen^®^, animals received 1, 5, or 10 mg/kg/day (designated as LOW, MID, and HIGH, respectively) for 14 consecutive days following SCP induction. Because inhalational anesthesia can influence tear secretion, all Schirmer measurements were performed using the same isoflurane anesthesia protocol at approximately the same time of day under standardized environmental conditions for all experimental groups. Therefore, although anesthesia may have influenced the absolute tear secretion values, its effect was expected to be comparable among groups and thus unlikely to affect the relative treatment comparisons. Lute-gen^®^, at all tested doses, improved SCP-induced reductions in tear secretion, accompanied by improvements in inflammatory and OS-related molecular markers. Lute-gen^®^ treatment was also associated with reduced expression of TLR4/MyD88/NF-κB signaling-related proteins, decreased MAPK phosphorylation, reduced MMP-2 and MMP-9 expression, and increased expression of Nrf2/HO-1-associated antioxidant markers and aquaporins (AQP1, AQP3, and AQP5) in corneal tissues. These findings are consistent with previous reports demonstrating the efficacy of bioactive compounds in mitigating DED-associated pathology in various in vivo models [38,48,49]. Collectively, these findings suggest that Lute-gen^®^ treatment was associated with improvements in tear homeostasis accompanied by modulation of inflammatory, antioxidant, and aquaporin-related molecular markers. Although these observations support a potential relationship between Lute-gen^®^ treatment and these signaling pathways, additional mechanistic studies employing pathway-specific inhibition or genetic approaches will be required to establish direct causal regulation.

Corneal fluorescein staining (CFS) is a widely used indicator of ocular surface epithelial damage and tear film instability in DED. In the present study, SCP administration resulted in a marked increase in CFS scores, reflecting significant corneal epithelial disruption and compromised barrier integrity. Treatment with Lute-gen^®^ significantly reduced CFS scores at all tested doses, indicating improved corneal epithelial integrity. This improvement was accompanied by a reduced expression of inflammatory and OS-related markers observed in the present study and is consistent with a previous study demonstrating the protective effects of cevimeline on ocular surface damage [50].

Conjunctival goblet cells are major contributors to maintaining the mucin layer of the tear film, and their loss is strongly associated with tear film instability and increased susceptibility to ocular surface damage in dry eye [51,52,53]. The recovery of PAS-positive goblet cells and mucin staining following Lute-gen^®^ treatment suggested restoration of this protective layer, and was consistent with a previous study demonstrating the protective effects of the ethanolic extract of Diospyros kaki in BAC-induced DED models [54]. Furthermore, lacrimal gland dysfunction is a major contributor to aqueous-deficient dry eye, and inflammatory alterations in this gland are tightly linked to reduced tear output [55,56]. Notably, Lute-gen^®^ treatment was associated with preservation of lacrimal gland architecture, as evidenced by improved acinar structure and cell density. These observations are consistent with a previous study reporting the protective effects of *Tagetes erecta* Linn. flower extract in a desiccation stress-induced DED mouse model [57].

MMP9 is a well-recognized mediator of ocular surface damage in DED and is closely associated with epithelial barrier disruption and disease severity [7,58]. In the present study, Lute-gen^®^ reduced MMP2 and MMP9 protein expression and decreased MMP9 immunofluorescence staining in both corneal and conjunctival tissues, indicating that the molecular changes observed in the present study were accompanied by reduced tissue-level inflammatory alterations. In addition, the immunofluorescence analysis demonstrated reduced TLR4 expression together with decreased CD68^+^ inflammatory cell infiltration. This is important because epithelial–immune cell interaction is a core feature of dry eye inflammation, and immune cell recruitment amplifies ocular surface damage once the barrier is disturbed [59,60]. Macrophage involvement in dry eye-associated ocular tissues, including the cornea and lacrimal gland, has been documented in previous studies [8,9,61,62]. Collectively, these findings suggest that Lute-gen^®^ treatment was associated with reduced inflammatory signaling and inflammatory cell infiltration in ocular surface tissues. The reductions in TLR4 expression and CD68^+^ inflammatory cell infiltration following Lute-gen^®^ treatment are consistent with previously reported inhibitory effects of the artemisinin analog SM934 in the SCP-induced DED model [38].

There are a few limitations associated with this study. The SCP-induced in vivo model represents acute/subacute aqueous-deficient dry eye, which captures important aspects of tear deficiency and inflammation but does not fully capture the clinical complexity of human disease [44,45]. In particular, this model does not fully reproduce other clinically important forms of dry eye disease, including evaporative dry eye associated with meibomian gland dysfunction, autoimmune-related lacrimal gland disease, or patient-reported symptom outcomes. Therefore, these findings should be interpreted within the context of this experimental model and require further validation in additional preclinical and clinical studies. In addition, the in vitro model used TNF-α stimulation to investigate inflammatory and oxidative responses. Although this model reproduces important inflammatory features of DED, it does not fully reflect the hyperosmolar stress component that plays a central role in disease initiation. Future studies using hyperosmolar stress alone or in combination with inflammatory stimuli would further strengthen the translational relevance of these findings. Moreover, although multiple signaling pathways were analyzed, direct inhibition studies were not performed to establish causal relationships. Finally, although significant improvements in tear secretion, conjunctival goblet cell preservation, lacrimal gland morphology, and ocular surface integrity were observed, molecular analyses were primarily performed using corneal tissue. Consequently, the molecular mechanisms underlying the protective effects of Lute-gen^®^ in the conjunctiva and lacrimal gland were not directly evaluated. Future studies incorporating tissue-specific molecular analyses of these ocular tissues will be valuable for further validating the mechanisms associated with the observed functional and histological improvements. Nevertheless, the consistency of the functional, histological, and molecular findings provides complementary evidence supporting the potential protective effects of Lute-gen^®^ in experimental dry eye disease.

Overall, the present findings suggest that Lute-gen^®^ treatment was associated with improvements across multiple features of experimental dry eye disease. Specifically, Lute-gen^®^ treatment was associated with enhanced antioxidant marker expression, reduced inflammatory responses and inflammatory cell infiltration, restoration of aquaporin expression, and preservation of ocular surface and lacrimal gland morphology. These findings suggest that Lute-gen^®^ may exert protective effects across multiple aspects of experimental DED, where therapeutic approaches addressing multiple pathological processes may offer advantages over interventions directed at a single pathway [3].

## 5. Conclusions

In conclusion, Lute-gen^®^ showed significant protective effects against DED in both TNF-α-stimulated HCE-T cells and the SCP-induced rat model. Oral administration of Lute-gen^®^ improved epithelial cell survival and was associated with enhanced antioxidant marker expression, reduced expression of TLR4/NF-κB/NLRP3- and MAPK/MMP-related inflammatory markers, restoration of aquaporin expression, reduced CD68-positive inflammatory cell infiltration, and improvements in tear secretion and ocular tissue integrity. Taken together, these findings suggest that Lute-gen^®^ treatment was associated with improvements in experimental dry eye disease accompanied by modulation of antioxidant-, inflammatory-, matrix remodeling-, and aquaporin-related molecular markers. While these findings provide important preclinical evidence supporting the potential of Lute-gen^®^ for dry eye disease, additional mechanistic studies employing pathway-specific inhibition or genetic approaches will be required to establish direct causal relationships between Lute-gen^®^ treatment and the signaling pathways investigated in this study.

## Figures and Tables

**Table 1:** List of forward and reverse primer sets used for PCR amplification of the target genes.

Target Gene	Forward Primer Sequence	Reverse Primer Sequence
Human		
Nrf2	5′-CACATCCAGTCAGAAACCAGTGG-3′	5′-GGAATGTCTGCGCCAAAAGCTG-3′
HO-1	5′-CCAGGCAGAGAATGCTGAGTTC-3′	5′-AAGACTGGGCTCTCCTTGTTGC-3′
SOD	5′-CTCACTCTCAGGAGACCATTGC-3′	5′-CCACAAGCCAAACGACTTCCAG-3′
CAT	5′-GTGCGGAGATTCAACACTGCCA-3′	5′-CGGCAATGTTCTCACACAGACG-3′
GPx	5′-GTGCTCGGCTTCCCGTGCAAC-3′	5′-CTCGAAGAGCATGAAGTTGGGC-3′
IL-1β	5′-CCACAGACCTTCCAGGAGAATG-3′	5′-GTGCAGTTCAGTGATCGTACAGG-3′
IL-6	5′-AGACAGCCACTCACCTCTTCAG-3′	5′-TTCTGCCAGTGCCTCTTTGCTG-3′
TNF-α	5′-CTCTTCTGCCTGCTGCACTTTG-3′	5′-ATGGGCTACAGGCTTGTCACTC-3′
AQP3	5′-GAGATGCTCCACATCCGCTAC-3′	5′-CACACGATAAGGGAGGCTGTGC-3′
AQP5	5′-TACGGTGTGGCACCGCTCAATG-3′	5′-AGTCAGTGGAGGCGAAGATGCA-3′
β-actin	5′-CACCATTGGCAATGAGCGGTTC-3′	5′-AGGTCTTTGCGGATGTCCACGT-3′
Rat		
Nrf2	5′-CCATTTACGGAGACCCAC-3′ -3′	5′-TGAGCGGCAACTTTATTC-3′
HO-1	5′-TGCTCGCATGAACACTCTG-3′	5′-TCCTCTGTCAGCAGTGCCT-3′
SOD	5′-GAAGGCCGTGTGCGTGCTG-3′	5′-GGACACATTGGCCACACCG-3′
CAT	5′-GCGAATGGAGAGGCAGTGTAC-3′	5′-GAGTGACGTTGTCTTCATTAGCACTG-3′
GPx	5′-CTCTCCGCGGTGGCACAGT-3′	5′-CCACCACCGGGTCGGACATAC-3′
IL-1β	5′-GACTTCACCATGGAACCCGT-3′	5′-GGAGACTGCCCATTCTCGAC-3′
IL-6	5′-AGCGATGATGCACTGTCAGA-3′	5′-GGAACTCCAGAAGACCAGAGC-3′
TNF-α	5′-CTGTGCCTCAGCCTCTTCTC-3′	5′-ACTGATGAGAGGGAGCCCAT-3′
TLR4	5′-AGCTTCTCCAATTTCTCACAACTTC-3′	5′-TGAGAGCTGGTTTAAGCCATGC-3′
MyD88	5′-CTGGGGCAAACGCCGGAGCT-3′	5′-ATGAGCTCGCTGGCGATGGA-3′
NLRP3	5′-GTGGAGATCCTAGGTTTCTCTG-3′	5′-CAGGATCTCATTCTCTTGGATC-3′
AQP1	5′-ACCTGCTGGCCATTGACTAC-3′	5′-CCAGGGCACTCCCAATGAAT-3′
AQP3	5′-GAGATGCTCCACATCCGCTAC-3′	5′-CACACAATAAGGGCTGCTGTGC-3′
AQP5	5′-TGTGCTCCCTTGCCTTCTTC-3′	5′-TGGCCCAGTGTGACAGACAA-3′
β-actin	5′-CTTGCAGCTCCTCCGTCGCC-3′	5′-CTTGCTCTGGGCCTCGTCGC-3′

Abbreviations: Nrf2, nuclear factor erythroid 2-related factor 2; HO-1, heme oxygenase-1; SOD, superoxide dismutase; CAT, catalase; GPx, glutathione peroxidase; IL, interleukin; TNF-α, tumor necrosis factor-alpha; AQP, aquaporin; TLR4, toll like receptor 4; MyD88, myeloid differentiation primary response 88; and NLRP3, NOD-, LRR- and pyrin domain-containing protein 3.

## Data Availability

The data supporting the findings of this study are available from the corresponding author upon reasonable request.

## Supplementary Materials

The following supporting information can be downloaded at https://www.mdpi.com/article/10.3390/antiox15070872/s1. Figure S1. Uncropped original Western blot images corresponding to Figure 3H–L; Figure S2. Uncropped original Western blot images corresponding to Figure 4E–H; Figure S3. Uncropped original Western blot images corresponding to Figure 5C–D; Figure S4. Effect of Lute-gen^®^ on food and water intake in SCP-induced dry eye rats; Figure S5. Uncropped original Western blot images corresponding to Figure 8F–J; Figure S6. Uncropped original Western blot images corresponding to Figure 9G–N; Figure S7. Different-magnification representative immunofluorescence images of TLR4 expression in conjunctival tissues; Figure S8. Different-magnification representative immunofluorescence images of CD68 expression in conjunctival tissues; Figure S9. Different-magnification representative immunofluorescence images of MMP9 expression in conjunctival tissues; Figure S10. Different-magnification representative immunofluorescence images of TLR4 expression in corneal tissues; Figure S11. Different-magnification representative immunofluorescence images of MMP9 expression in corneal tissues; Figure S12. Uncropped original Western blot images corresponding to Figure 10D–F; Source Data S1: In vitro Western blot densitometric calculations; Source Data S2: In vivo Western blot densitometric calculations.
