# Supplementary material for: Lute-Gen® Alleviates Dry Eye Disease and Modulates Nrf2/HO-1, TLR4/NF-κB/MAPK Signaling, and Aquaporin-Mediated Tear Homeostasis
Source: Antioxidants (Basel). 2026 Jul 13;15(7):872. doi: 10.3390/antiox15070872 (PMC13406100; doi:10.3390/antiox15070872)
Supplement: Supplementary file 1 [file antioxidants-15-00872-s001.zip › Supplementary Information.pdf]

Supplementary Information

Lute-Gen® Alleviates Dry Eye Disease and Modulates Nrf2/HO-1, TLR4/NF-κB/MAPK Signaling, and Aquaporin-Mediated Tear Homeostasis

Rachit Sood [1,2], Sanjay [2,3,*] and Hae-Jeung Lee [1,2,3,4,*]

[1] Department of Health Sciences and Technology, Gachon Advanced Institute for Health Science and Technology (GAIHST), Gachon University, Incheon 21999, Republic of Korea; soodrachit@gachon.ac.kr or rachitsood1998@gmail.com
[2] Institute for Aging and Clinical Nutrition Research, Gachon University, Seongnam 13120, Republic of Korea
[3] Department of Food and Nutrition, College of BioNano Technology, Gachon University, Seongnam 13120, Republic of Korea
[4] Gachon Biomedical Convergence Institute, Gachon University Gil Medical Center, Incheon 21565, Republic of Korea
* Correspondence: san55@gachon.ac.kr or sanjay.monga4@gmail.com (S.); skysea@gachon.ac.kr or skysea1010@gmail.com (H.-J.L.); Tel.: +82-31-750-5968 (H.-J.L.)

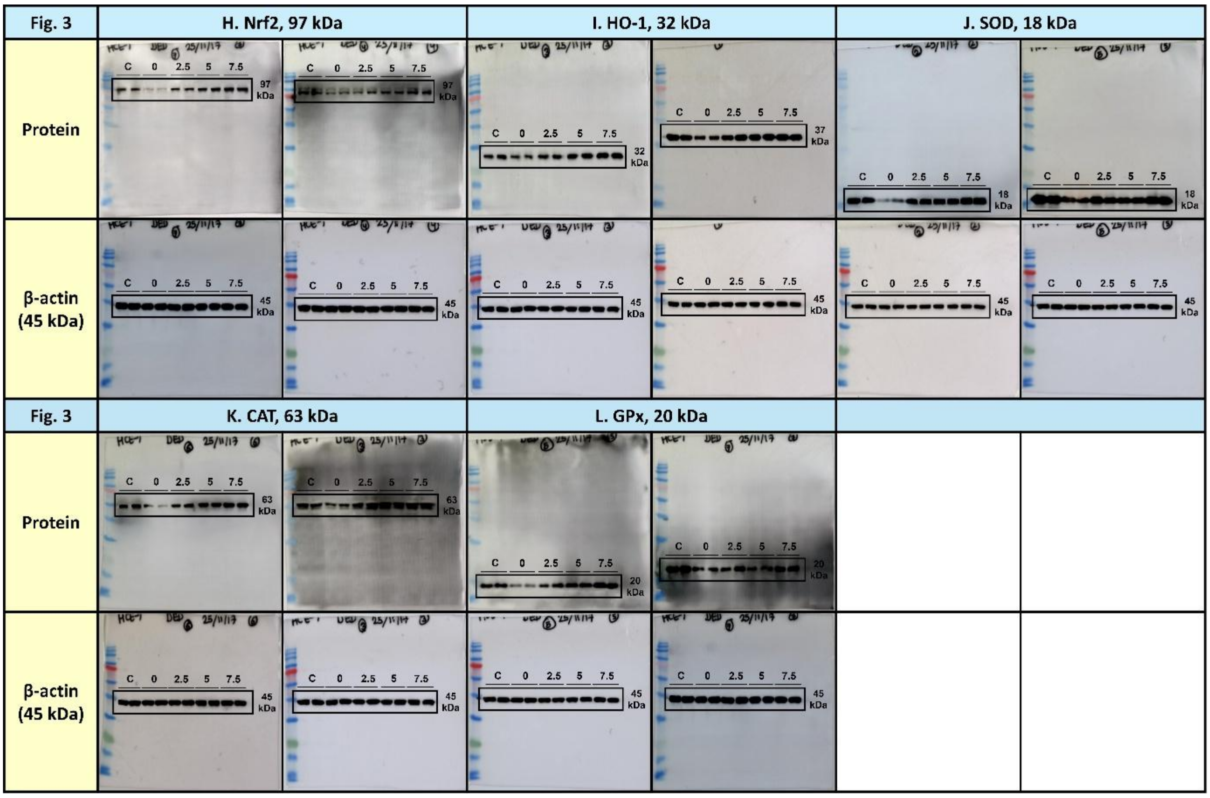

Figure S1. Uncropped original western blot images corresponding to Figure 3H-L in the main manuscript. For each target protein, blots represent two biological replicates per group. The left blot for each target protein corresponds to the representative blot presented in the main manuscript, whereas the right blot represents an additional biological replicate. The representative blot shown in the main manuscript corresponds to one of the biological replicates included in the densitometric analysis, and all biological replicates were included in the quantitative analysis. All target proteins (Nrf2, HO-1, SOD, CAT, and GPx) were detected on independent membranes; therefore, each blot was normalized to its corresponding β-actin loading control obtained from the same membrane. As stated in the manuscript, n = 3 for each group. All full-length blots are provided here for transparency and data integrity.

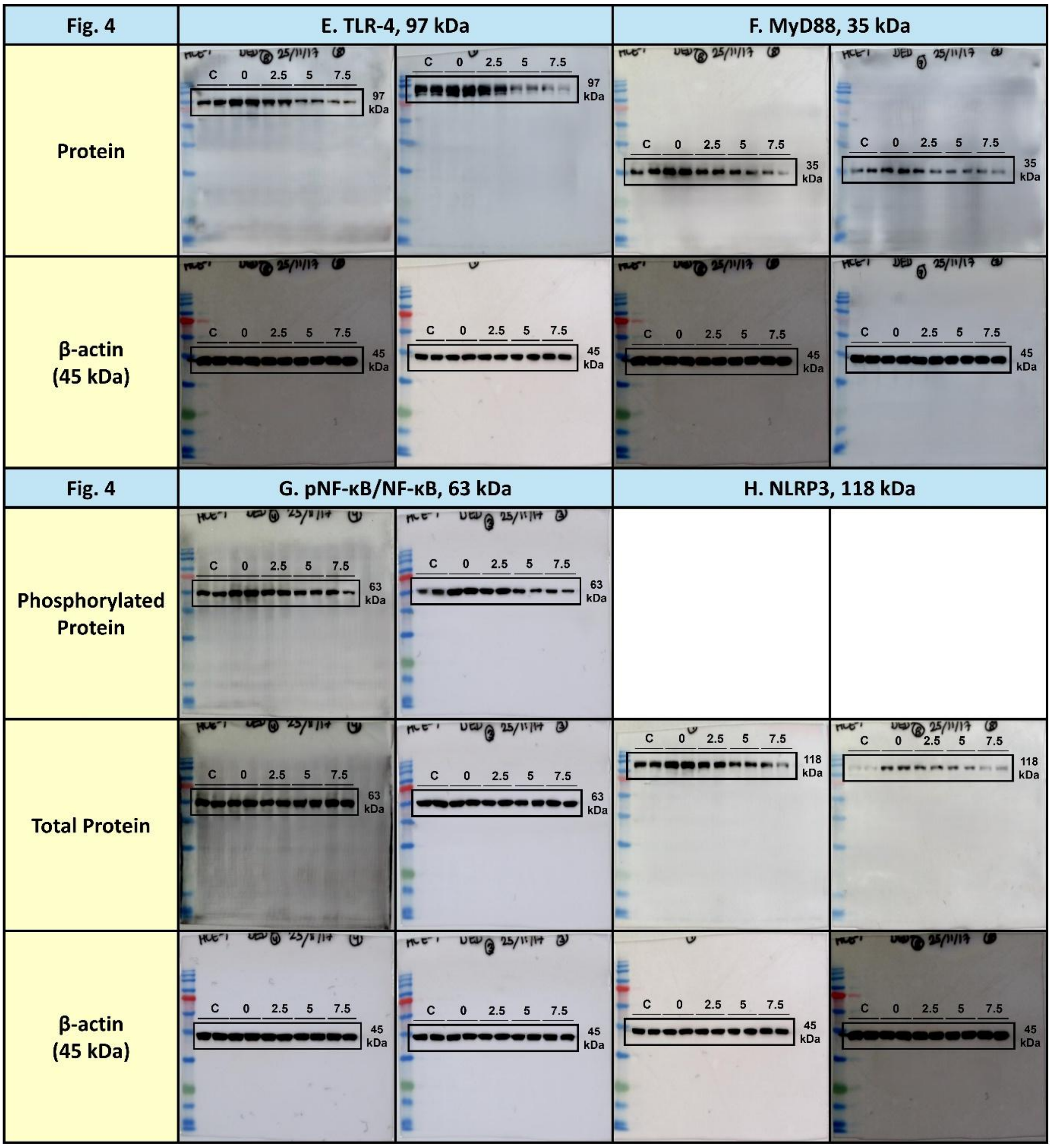

Figure S2. Uncropped original western blot images corresponding to Figure 4E-H in the main manuscript. For each target protein, blots represent two biological replicates per group. The left blot for each target protein corresponds to the representative blot presented in the main manuscript, whereas the right blot represents an additional biological replicate. The representative blot shown in the main manuscript corresponds to one of the biological replicates included in the densitometric analysis, and all biological replicates were included in the quantitative analysis. In the representative blots, TLR4 and MyD88 were sequentially detected on the same membrane following stripping and reprobing and therefore share the same β-actin loading control. The additional biological replicate blots were detected on independent membranes; therefore, each blot was normalized to its corresponding β-actin loading control. As stated in the manuscript, n = 3 for each group. All full-length blots are provided here for transparency and data integrity.

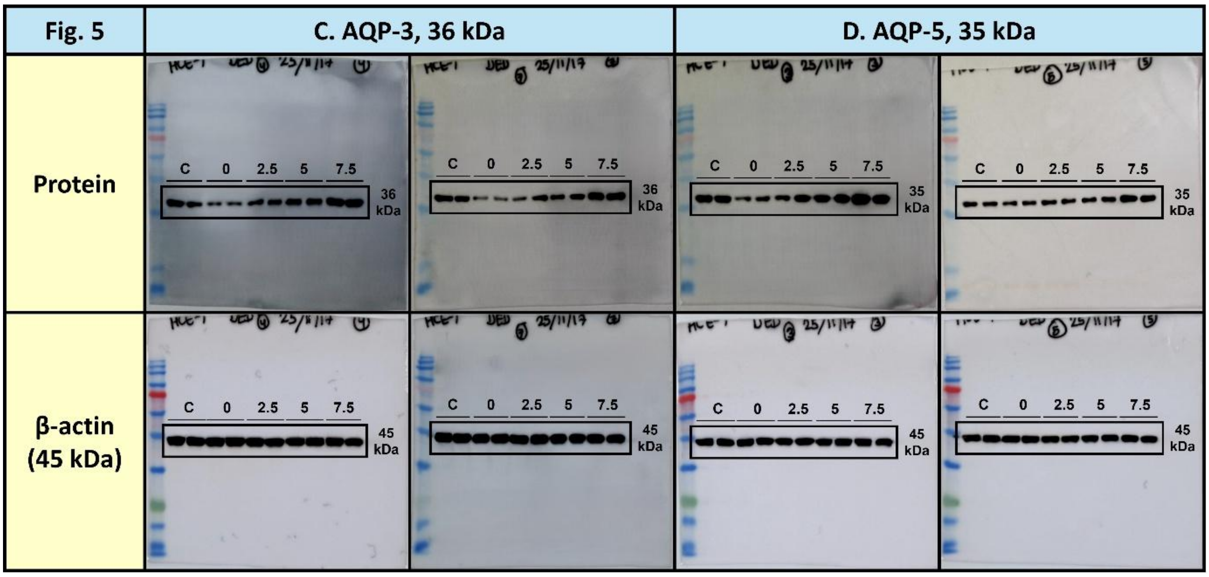

Figure S3. Uncropped original western blot images corresponding to Figure 5C-D in the main manuscript. For each target protein, blots represent two biological replicates per group. The left blot for each target protein corresponds to the representative blot presented in the main manuscript, whereas the right blot represents an additional biological replicate. The representative blot shown in the main manuscript corresponds to one of the biological replicates included in the densitometric analysis, and all biological replicates were included in the quantitative analysis. Both the representative and additional biological replicate blots for AQP3 and AQP5 were detected on independent membranes; therefore, each blot was normalized to its corresponding β-actin loading control obtained from the same membrane. As stated in the manuscript, n = 3 for each group. All full-length blots are provided here for transparency and data integrity.

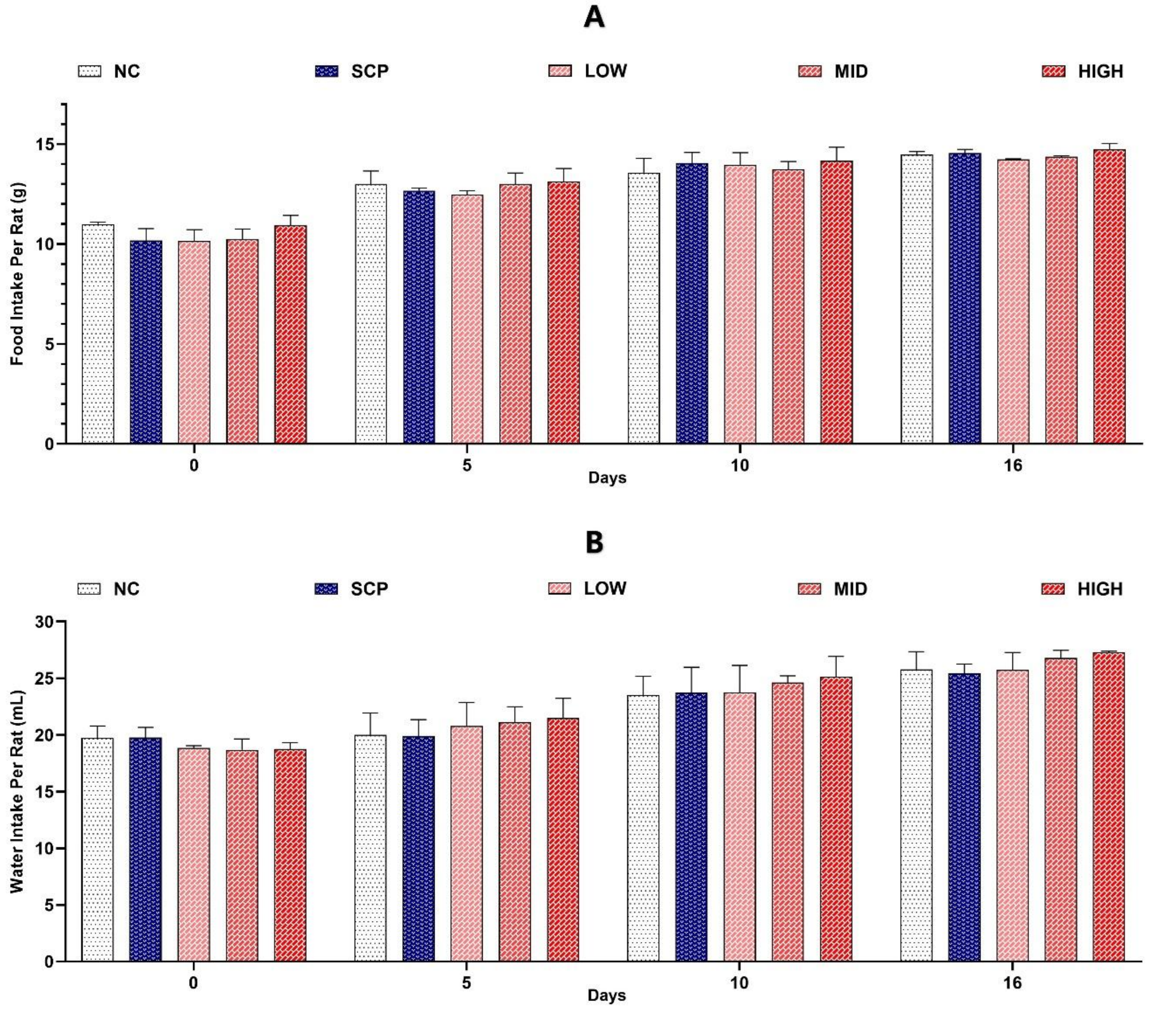

Figure S4. Effect of Lute-gen® on food and water intake in SCP-induced dry eye rats. (A) Food intake and (B) water intake during the experimental period. Data are presented as mean ± SD (n = 10 animals per group). NC, normal control; SCP, scopolamine-treated group; LOW, Lute-gen® (1 mg/kg); MID, Lute-gen® (5 mg/kg); and HIGH, Lute-gen® (10 mg/kg).

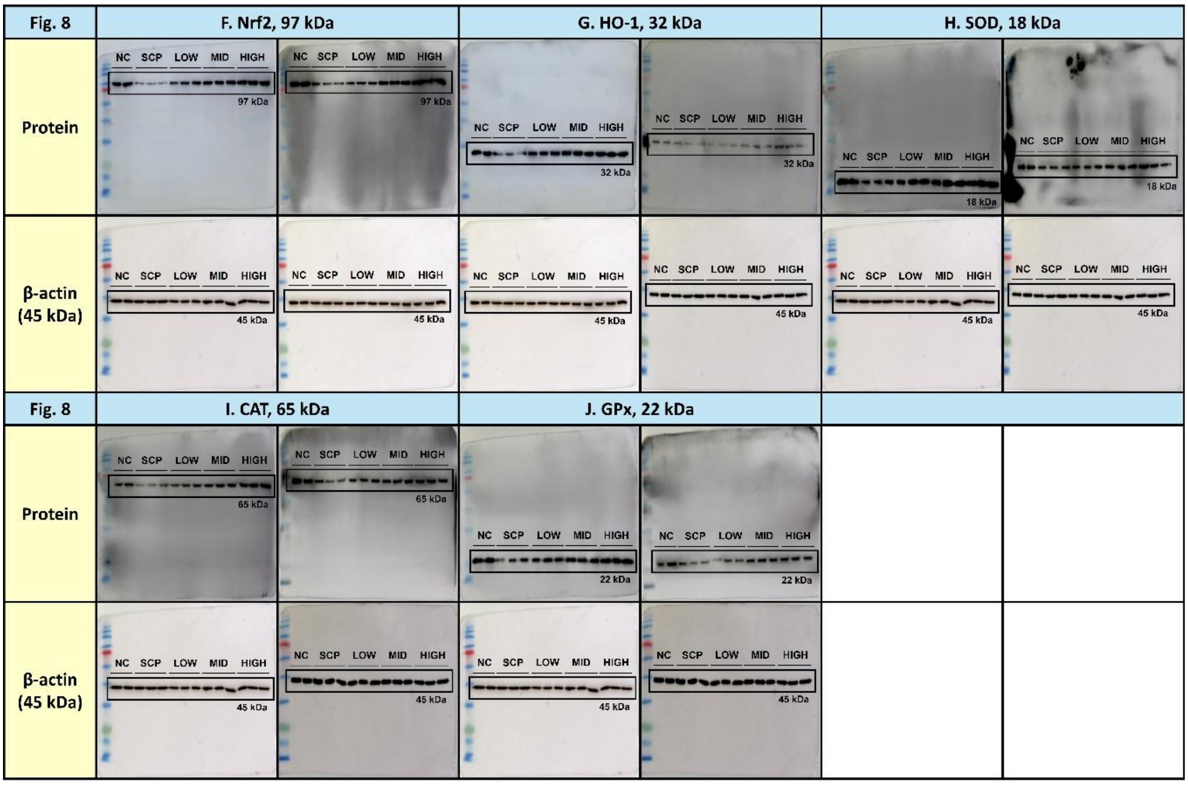

Figure S5. Uncropped original western blot images corresponding to Figure 8F-J in the main manuscript. For each target protein, blots represent three biological replicates per group (except NC: 2). The left blot for each target protein corresponds to the representative blot presented in the main manuscript, whereas the remaining blots represent additional biological replicates. The representative blot shown in the main manuscript corresponds to one of the biological replicates included in the densitometric analysis, and all biological replicates were included in the quantitative analysis. In the representative blots, Nrf2, SOD, CAT, and GPx were sequentially detected on the same membrane following stripping and reprobing and therefore share the same β-actin loading control, whereas HO-1 was detected on a separate membrane. Among the additional biological replicates, CAT and GPx were sequentially detected on the same membrane, and HO-1 and SOD were sequentially detected on another membrane, while Nrf2 was detected on an independent membrane. Accordingly, proteins detected on the same membrane share the corresponding β-actin loading control, whereas proteins analyzed on separate membranes were normalized to their respective β-actin controls. As stated in the manuscript, n = 6 animals for each group. All full-length blots are provided here for transparency and data integrity.

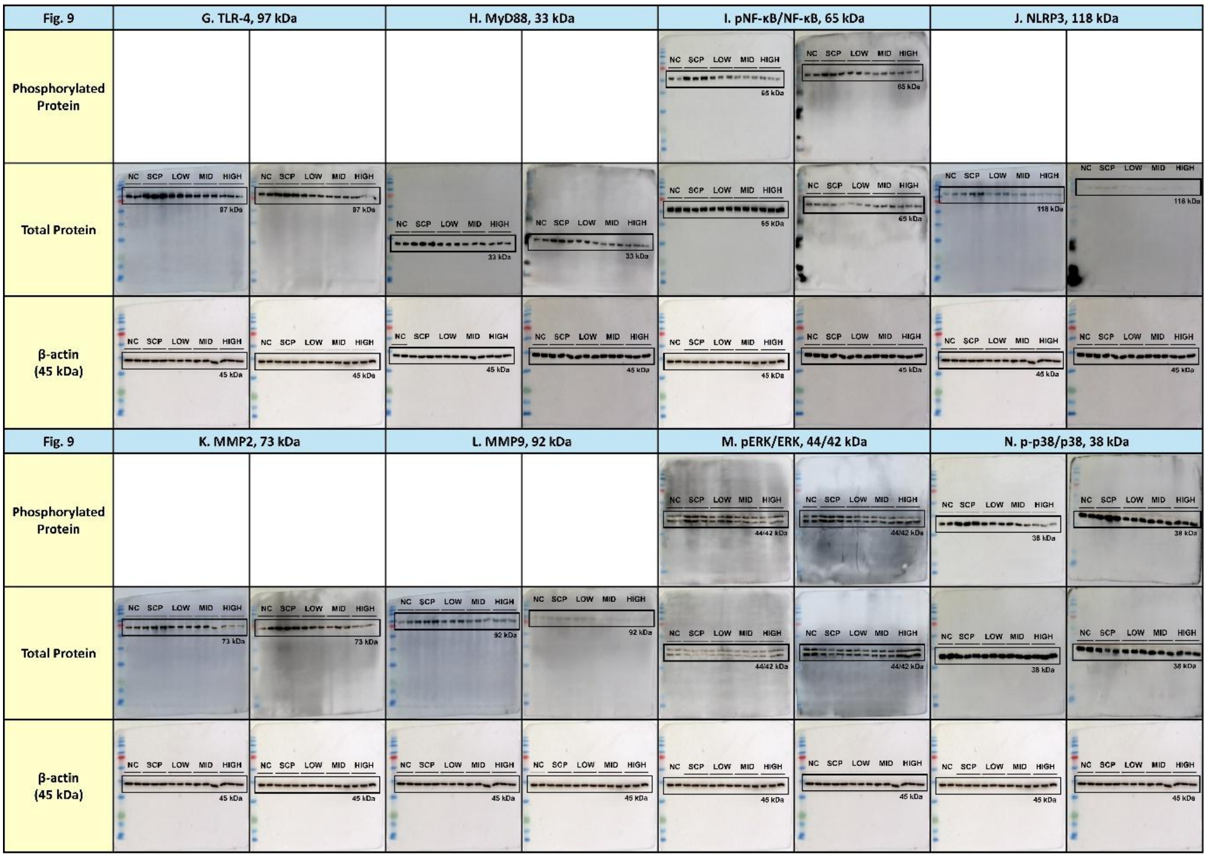

Figure S6. Uncropped original western blot images corresponding to Figure 9G-N in the main manuscript. For each target protein, blots represent three biological replicates per group (except NC: 2). The left blot for each target protein corresponds to the representative blot presented in the main manuscript, whereas the remaining blots represent additional biological replicates. The representative blot shown in the main manuscript corresponds to one of the biological replicates included in the densitometric analysis, and all biological replicates were included in the quantitative analysis. In the representative blots, TLR4, NLRP3, MMP2, and MMP9 were sequentially detected on the same membrane following stripping and reprobing and therefore share the same β-actin loading control, while p-NF-κB/NF-κB and p-ERK/ERK and p-p38/p38 were each detected on separate membranes using phosphorylation-specific normalization to their corresponding total proteins. Among the additional biological replicates, TLR4, MMP2, and MMP9 were sequentially detected on the same membrane, MyD88, p-NF-κB/NF-κB, and NLRP3 were sequentially detected on another membrane, and p-ERK/ERK and p-p38/p38 were sequentially detected on a separate membrane. Accordingly, proteins detected on the same membrane share the corresponding β-actin loading control, whereas phosphorylated proteins were normalized to their respective total proteins. As stated in the manuscript, n = 6 animals for each group. All full-length blots are provided here for transparency and data integrity.

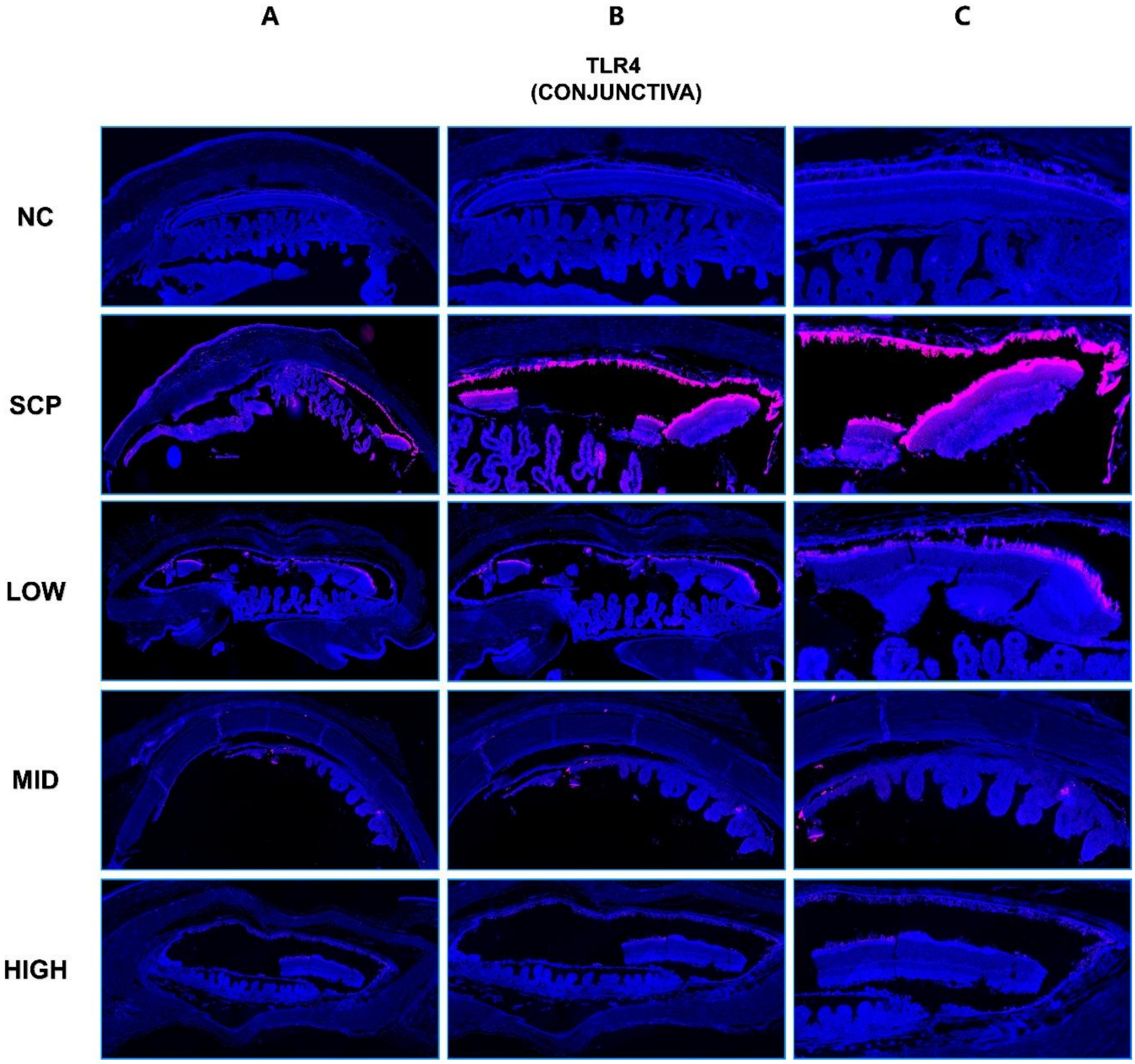

Figure S7. Different-magnification representative immunofluorescence images of TLR4 expression in conjunctival tissues. (A-C) Representative immunofluorescence images showing low-, medium-, and high-magnification views of TLR4 expression in conjunctival tissues. Scale bars = 100 μm (A), 50 μm (B), and 10 μm (C). NC, normal control; SCP, scopolamine-treated group; LOW, Lute-gen® (1 mg/kg); MID, Lute-gen® (5 mg/kg); and HIGH, Lute-gen® (10 mg/kg).

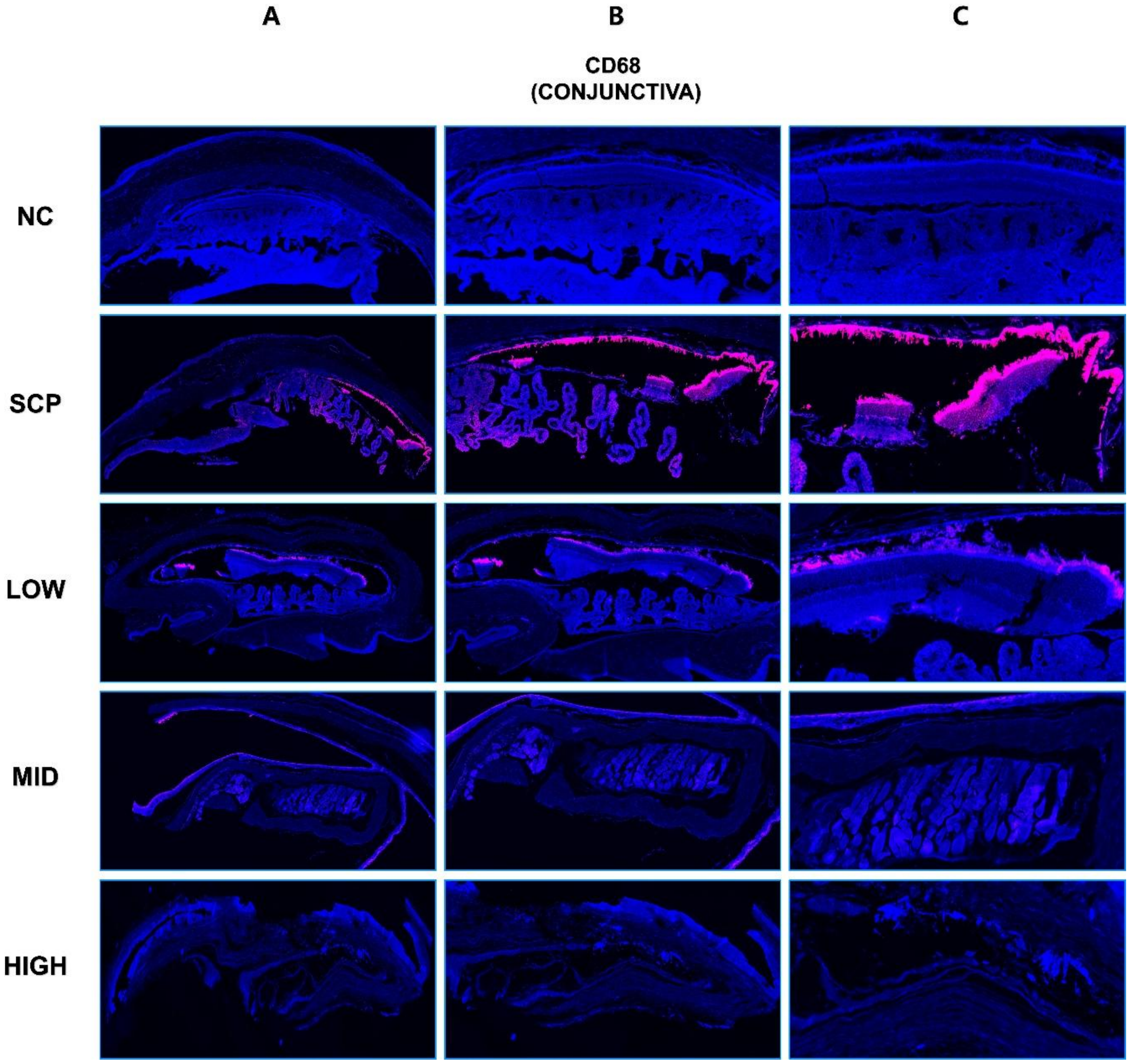

Figure S8. Different-magnification representative immunofluorescence images of CD68 expression in conjunctival tissues. (A-C) Representative immunofluorescence images showing low-, medium-, and high-magnification views of CD68 expression in conjunctival tissues. Scale bars = 100 μm (A), 50 μm (B), and 10 μm (C). NC, normal control; SCP, scopolamine-treated group; LOW, Lute-gen® (1 mg/kg); MID, Lute-gen® (5 mg/kg); and HIGH, Lute-gen® (10 mg/kg).

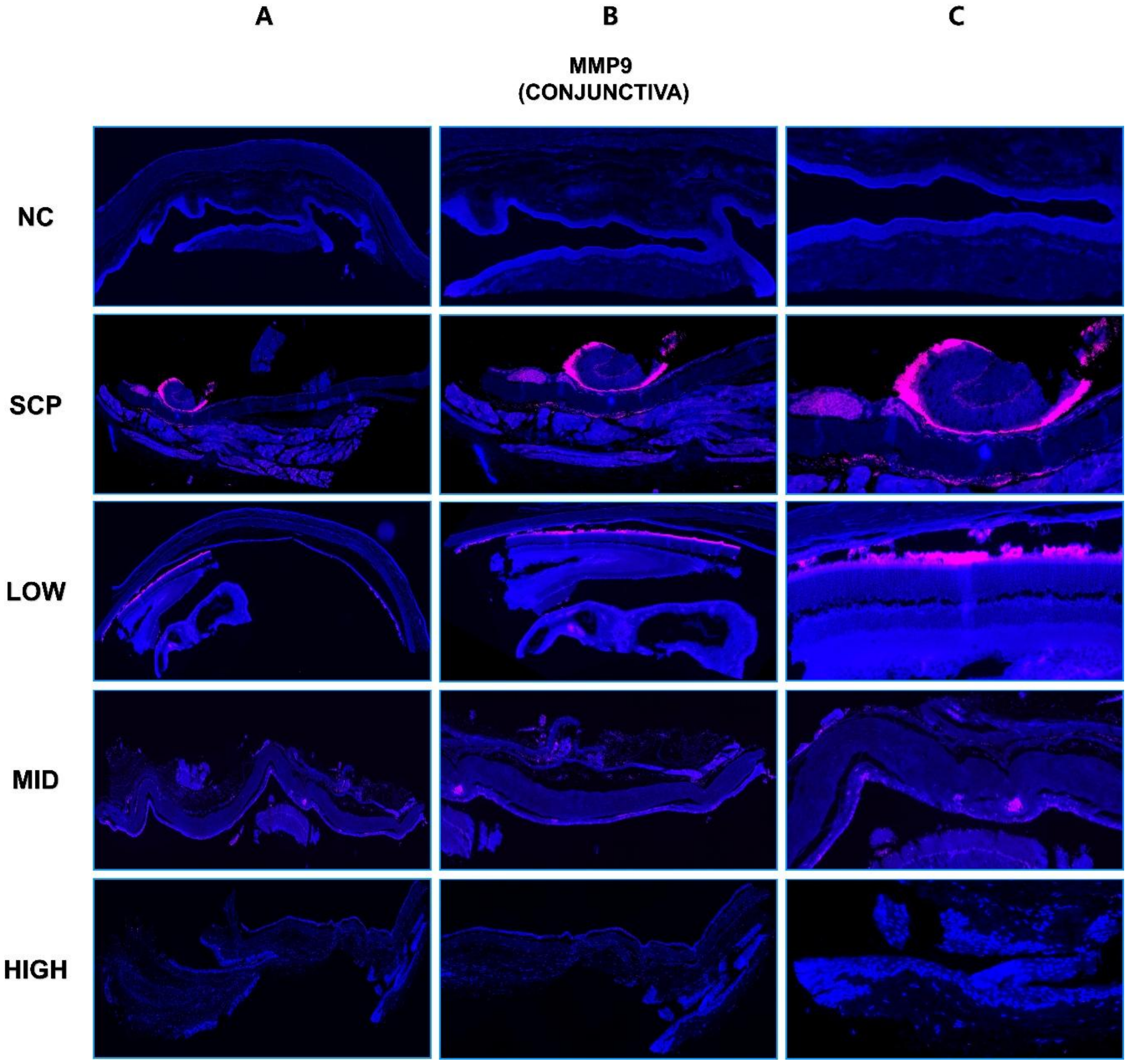

Figure S9. Different-magnification representative immunofluorescence images of MMP9 expression in conjunctival tissues. (A-C) Representative immunofluorescence images showing low-, medium-, and high-magnification views of MMP9 expression in conjunctival tissues. Scale bars = 100 μm (A), 50 μm (B), and 10 μm (C). NC, normal control; SCP, scopolamine-treated group; LOW, Lute-gen® (1 mg/kg); MID, Lute-gen® (5 mg/kg); and HIGH, Lute-gen® (10 mg/kg).

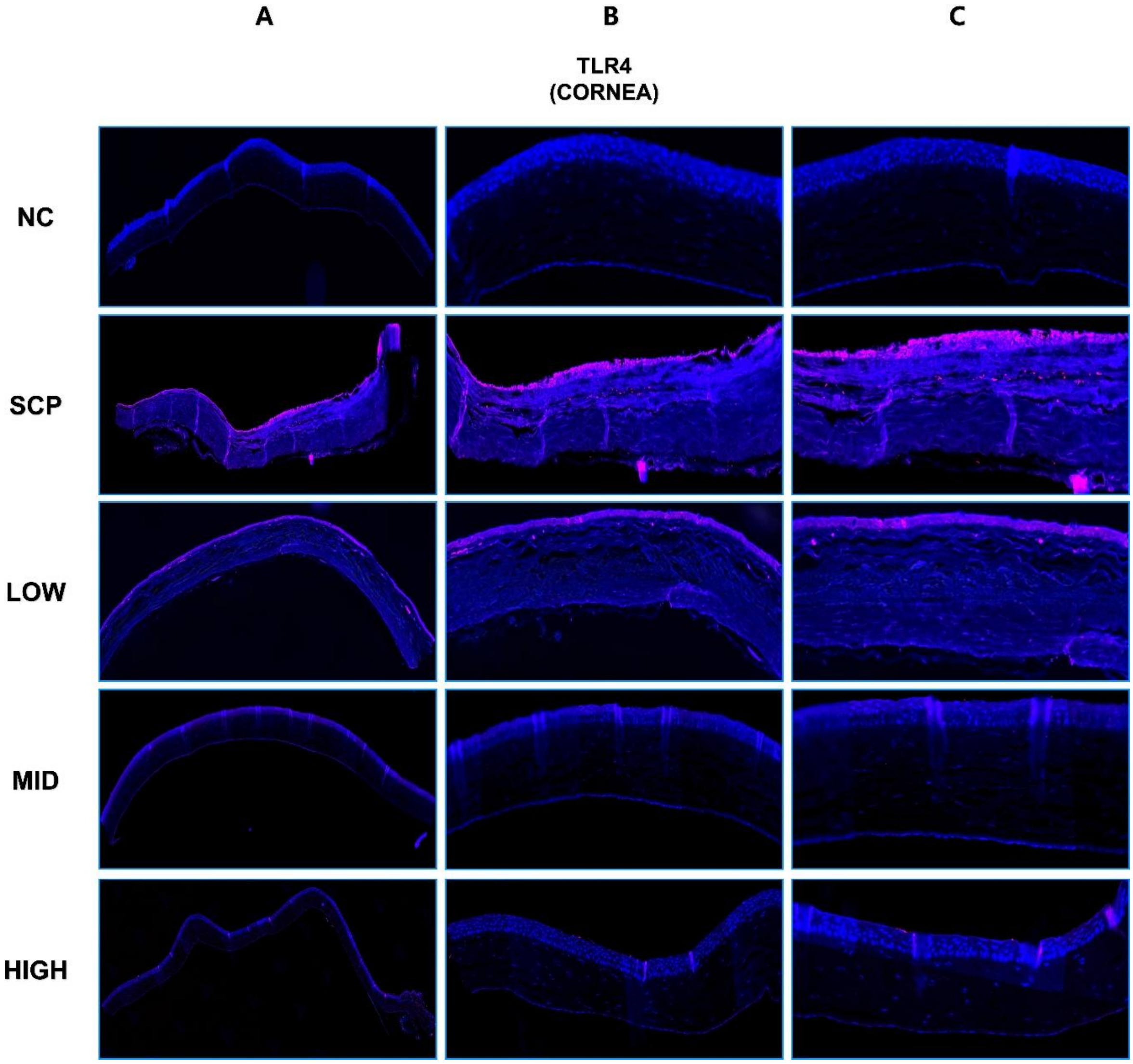

Figure S10. Different-magnification representative immunofluorescence images of TLR4 expression in corneal tissues. (A-C) Representative immunofluorescence images showing low-, medium-, and high-magnification views of TLR4 expression in corneal tissues. Scale bars = 100 μm (A), 50 μm (B), and 10 μm (C). NC, normal control; SCP, scopolamine-treated group; LOW, Lute-gen® (1 mg/kg); MID, Lute-gen® (5 mg/kg); and HIGH, Lute-gen® (10 mg/kg).

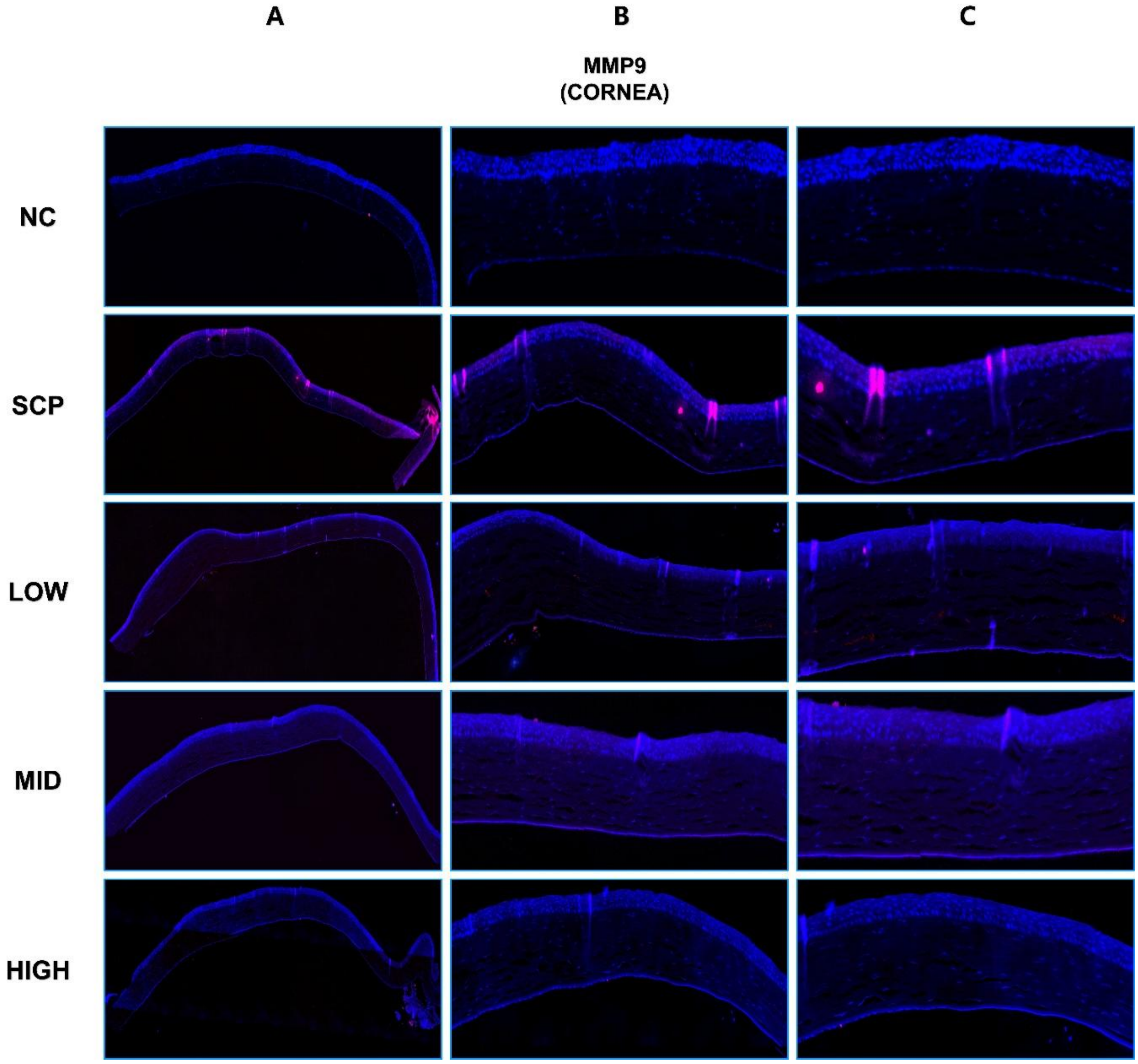

Figure S11. Different-magnification representative immunofluorescence images of MMP9 expression in corneal tissues. (A-C) Representative immunofluorescence showing low-, medium-, and high-magnification views of MMP9 expression in corneal tissues. Scale bars = 100 μm (A), 50 μm (B), and 10 μm (C). NC, normal control; SCP, scopolamine-treated group; LOW, Lute-gen® (1 mg/kg); MID, Lute-gen® (5 mg/kg); and HIGH, Lute-gen® (10 mg/kg).

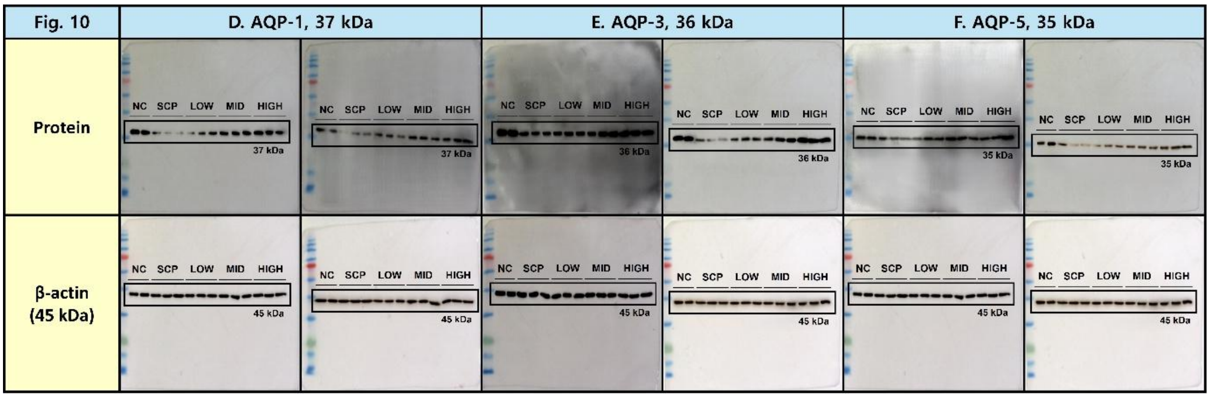

Figure S12. Uncropped original western blot images corresponding to Figure 10D-F in the main manuscript. For each target protein, blots represent three biological replicates per group (except NC: 2). The left blot for each target protein corresponds to the representative blot presented in the main manuscript, whereas the remaining blots represent additional biological replicates. The representative blot shown in the main manuscript corresponds to one of the biological replicates included in the densitometric analysis, and all biological replicates were included in the quantitative analysis. In the representative blots, AQP1 and AQP5 were sequentially detected on the same membrane following stripping and reprobing and therefore share the same β-actin loading control, whereas AQP3 was detected on a separate membrane. Among the additional biological replicates, AQP3 and AQP5 were sequentially detected on the same membrane, while AQP1 was detected on an independent membrane. Accordingly, proteins detected on the same membrane share the corresponding β-actin loading control, whereas proteins analyzed on separate membranes were normalized to their respective β-actin controls. As stated in the manuscript, n = 6 animals for each group. All full-length blots are provided here for transparency and data integrity.
